# Cultivation, genomics, and giant viruses of a ubiquitous and heterotrophic freshwater cryptomonad

**DOI:** 10.1093/ismejo/wraf271

**Published:** 2025-12-06

**Authors:** Indranil Mukherjee, Paul-Adrian Bulzu, Roudaina Boukheloua, Usman Asghar, Hongjae Park, Helena Henriques Vieira, Maria-Cecilia Chiriac, Vojtěch Kasalický, Petr Znachor, Pavel Rychtecký, Karel Šimek, Michaela M Salcher, Markus Haber, Rohit Ghai

**Affiliations:** Biology Centre of the Czech Academy of Sciences, Institute of Hydrobiology, Na Sádkách 7, 37005 České Budějovice, Czech Republic; Biology Centre of the Czech Academy of Sciences, Institute of Hydrobiology, Na Sádkách 7, 37005 České Budějovice, Czech Republic; Biology Centre of the Czech Academy of Sciences, Institute of Hydrobiology, Na Sádkách 7, 37005 České Budějovice, Czech Republic; Faculty of Science, University of South Bohemia, 37005 České Budějovice, Czech Republic; Biology Centre of the Czech Academy of Sciences, Institute of Hydrobiology, Na Sádkách 7, 37005 České Budějovice, Czech Republic; Faculty of Science, University of South Bohemia, 37005 České Budějovice, Czech Republic; Biology Centre of the Czech Academy of Sciences, Institute of Hydrobiology, Na Sádkách 7, 37005 České Budějovice, Czech Republic; Department of Biological Sciences and Bioengineering, Inha University, 22212, Incheon, Republic of Korea; Biology Centre of the Czech Academy of Sciences, Institute of Hydrobiology, Na Sádkách 7, 37005 České Budějovice, Czech Republic; Biology Centre of the Czech Academy of Sciences, Institute of Hydrobiology, Na Sádkách 7, 37005 České Budějovice, Czech Republic; Biology Centre of the Czech Academy of Sciences, Institute of Hydrobiology, Na Sádkách 7, 37005 České Budějovice, Czech Republic; Biology Centre of the Czech Academy of Sciences, Institute of Hydrobiology, Na Sádkách 7, 37005 České Budějovice, Czech Republic; Faculty of Science, University of South Bohemia, 37005 České Budějovice, Czech Republic; Biology Centre of the Czech Academy of Sciences, Institute of Hydrobiology, Na Sádkách 7, 37005 České Budějovice, Czech Republic; Biology Centre of the Czech Academy of Sciences, Institute of Hydrobiology, Na Sádkách 7, 37005 České Budějovice, Czech Republic; Biology Centre of the Czech Academy of Sciences, Institute of Hydrobiology, Na Sádkách 7, 37005 České Budějovice, Czech Republic; Biology Centre of the Czech Academy of Sciences, Institute of Hydrobiology, Na Sádkách 7, 37005 České Budějovice, Czech Republic; Biology Centre of the Czech Academy of Sciences, Institute of Hydrobiology, Na Sádkách 7, 37005 České Budějovice, Czech Republic

**Keywords:** cryptophytes, cryptomonads, flagellates, aquatic food web, freshwaters, biogeography, cultivation, CARD-FISH, whole-genome sequencing, giant virus

## Abstract

Heterotrophic nanoflagellates are the chief agents of bacterivory in the aquatic microbial loop but remain underrepresented in culture collections and in genomic databases. We isolated and characterized a representative of the previously uncultured freshwater Cryptomonad Group 1 (CRY1a) lineage using a genome-streamlined, ultra-small and abundant microbe *Planktophila versatilis* as a prey and Catalyzed Reporter Deposition-Fluorescence in situ Hybridization (CARD-FISH) probe–based screening. This isolate, *Tyrannomonas regina*, is one of the most dominant ubiquitous heterotrophic cryptomonads in freshwaters. It is a small heterotrophic nanoflagellate (ca. 3–5 μm) and has the smallest genome of any cryptomonad sequenced thus far. The compact genome (ca. 69 Mb) revealed no traces of a photosynthetic lifestyle, consistent with its phylogenomic placement as a sister clade to cryptophytes that are characterized by the acquisition of a red-algal symbiont. Moreover, in comparison to its photosynthetic counterparts, its genome presents substantially lower repeat content and endogenous viral elements. Genomes of two giant viruses, *Tyrannovirus reginensis* GV1 and GV2, were also recovered from the same culture and represent a viral genus that has been described so far solely by metagenome-recovered genomes. Collectively, these findings provide insights into genomic ancestry and evolution, widespread ecological impact, and interactions of an elusive protist lineage and illustrate the advantages of culture-centric approaches towards unfolding complex tapestries of life in the microbial world.

## Introduction

Heterotrophic unicellular eukaryotes devour roughly a third of prokaryotic populations in aquatic habitats every day [1, 2]. The scale of this extermination is only matched by phages, which account for another third [2]. Considering the vast populations of prokaryotes in freshwaters, frequently in several millions per millilitre, this astonishing feat is achieved by engineering a high grazing rate, each heterotrophic flagellate ingesting 10–20 prokaryotes per hour [1, 3]. These flagellated bacterivores originate from diverse branches of the eukaryotic tree of life, including chrysophytes (a subgroup of Stramenopiles), telonemids, kinetoplastids, diplonemids, katablepharids, and cryptomonads [4]. In particular, heterotrophic nanoflagellates (HNF) (size ca. 3–5 μm) belonging to the cryptomonad Group 1 (CRY1a) lineage have emerged as one of the major bacterivorous agents in freshwater habitats [3, 5].

Cryptophytes, like many other plastid-bearing eukaryotes, have a secondary endosymbiotic event in their evolutionary history, when a red-algal cell became resident in the host cytoplasm, allowing the host cell to gain the ability to fix carbon [6]. However, unlike most other algae, there are four genomes in a single photosynthetic cryptophyte cell: the nuclear and mitochondrial genomes of the host, and the relict endosymbiont nucleus (or nucleomorph) and plastid genomes of the endosymbiont [7]. Only chlorarachniophytes and some dinoflagellates show similar persistence of the secondary endosymbiont nucleus, with the difference that the endosymbionts are derived from green algae [7, 8]. Such cryptophytes, like *Cryptomonas* and *Rhodomonas*, have been studied for nearly two centuries owing to their widespread distribution, recurrent bloom forming tendencies, and ease of isolation from freshwaters. The original isolation report of cryptomonads dates from the early nineteenth century and describes both *Cryptomonas* (photosynthetic) and *Chilomonas* (heterotrophic) [9]. However, it turned out that *Chilomonas* is secondarily heterotrophic owing to the loss of several photosynthesis genes [10] and leading to it having a leucoplast. Only a few decades later, another heterotrophic cryptomonad was isolated and called *Monas truncata* [11] and is now known as *Goniomonas truncata* [12]. Multiple relatives have been described since then, forming a freshwater and a marine lineage united as the order Goniomonadida and spanning multiple genera according to a recent revision [13]. Genomic analyses of a representative marine goniomonad, *Neptunogoniomonas avonlea* (formerly *Goniomonas avonlea*), have revealed that the group is ancestrally non-photosynthetic [14] and there was never an intrusion of a photosynthetic red alga in this lineage as is the case for all cryptophytes. Even though additional goniomonads continue to be described [5, 15], it is the closely related CRY1a lineage that appears to be far more abundant in freshwaters [5].

The CRY1 lineage remained obscure within the morphologically homogeneous “guild” of heterotrophic nanoflagellates until phylogenetic analyses of 18S rRNA gene sequences revealed a distinct clade related to goniomonads and as a deep branching lineage within an ancient origin within the cryptomonads [16]. The lineage was further split into CRY1a (freshwater) and CRY1b (marine) in a subsequent work when a representative of the marine CRY1b lineage, *Hemiarma marina*, was isolated using contaminant bacteria as a food source [17]. Direct quantitative observations were obtained from a brackish lagoon by using specific CARD-FISH probes targeting the 18S rRNA gene sequences of the CRY1a lineage [18]. Cells hybridized with the probe were small, aplastidic, and proposed to be bacterivores. CRY1a also appeared to be abundant members of the microbial community in the brackish lagoon where the study was conducted (17%–75% of all cryptomonads and 18%–100% of aplastidic flagellates). The freshwater lineage was also shown to be bacterivorous in enrichment experiments using specific CARD-FISH probes targeting both CRY1a and specific bacterial prey simultaneously [3]. CRY1a population increases were generally more pronounced with smaller prey, such as *Limnohabitans* Rim11, a short rod ca. 0.051 μm^3^ in volume. Multiple CARD-FISH–based comparative enumerations of bacterivorous protist lineages (CRY1a, katablepharids, telonemids) have revealed CRY1a to be by far the most abundant [1, 5, 19, 20]. Some estimates also suggest that the freshwater CRY1a lineage is more abundant in freshwaters than the marine CRY1b is in the marine habitat [5]. Taken together, even though the ecological role and scale of CRY1a as a major bacterivorous heterotrophic nanoflagellate have been well established, the lack of isolates impedes further experimental and genomic investigations.

We initiated this work to isolate and sequence one of the most abundant and ubiquitous bacterivorous flagellates in freshwater habitats. We reasoned that such an abundant but small nanoflagellate must necessarily consume the widespread genome-streamlined *Planktophila versatilis* (Actinomycetota, acI lineage) that is available in culture [21], without getting overwhelmed by other known fast-growing flagellates (*Spumella-*like chrysophytes) that are frequently recovered from cultivation-based methods for heterotrophic flagellates [22]. These ultra-small bacteria of the acI lineage have been considered to be less favoured by flagellates but still consumed [23]. We expected the generally faster-growing flagellates to display a slower growth response when confronted with ultra-small bacteria, as they appear to prefer larger prey. The use of an ultra-small prey coupled with extensive CARD-FISH–based screening resulted in the identification of a pure culture of *Tyrannomonas regina* (Greek for “Tyrant Queen”), an archetypal representative of the freshwater CRY1a lineage. We generated a high-quality draft genome sequence of this isolate, the smallest described for any cryptomonad as yet. No plastid or nucleomorph genomes were recovered from the genome assembly, reiterating its heterotrophic nature. Comparative genomics with other available cryptomonad genomes also revealed that endogenous polinton-like viruses (PLVs) in the *T. regina* genome are phylogenetically distinct from those seen in cryptophytes [24]. The genome sequencing also yielded two complete viral genomes that are the first representatives of giant viruses infecting heterotrophic cryptomonads. These findings provide fundamental and broad-based insights into the genomic, ecological, and evolutionary characteristics of one of the most dominant bacterivorous protists in freshwaters.

## Materials and methods

### Study sites and field sampling of freshwater habitats

Samples were collected from 87 lakes across 6 continents (Europe, Asia, Australia, Africa, North America, and South America) representing different trophic states, depths, areas, mixis types, and elevations (Supplementary Table S1). From each lake, samples were mainly collected from two to three depths (epilimnion, mid-water column, and hypolimnion) except for shallow lakes, where samples were collected from mid-water column depth. For several lakes, sampling was conducted over two seasons (spring and summer). Samples were also collected from a high-frequency sampling campaign (three times a week) at the Řimov Reservoir (Czech Republic) during Spring 2024. Transect sampling was conducted from some of the large lakes (Malawi and Balaton) to cover different parts of these lakes. The details of the samples collected from all the lakes and physicochemical parameters are provided in Supplementary Table S1. Enumeration of protists and CARD-FISH analyses with probe Cry1-652 [3] were performed as described before [20], and details are provided in Supplementary Information.

### Dilution-to-extinction cultivation, screening, and isolation of CRY1a

Water collected from 0.5-m depth of Řimov Reservoir (23 July 2021) was pre-filtered through 10-μm membrane filters to remove large eukaryotes. One millilitre of filtrate was added to five tubes containing 8 ml of filtered (0.2 μm) and autoclaved water collected from the reservoir and 1 ml of *Planktophila versatilis*, strain MsE-18 culture (ca. 2 × 10^7^ cells ml^−1^) as prey. After mixing, 1 ml from each tube was used as inoculum for a second tube (1:100 dilution of the original inoculum) that, in turn, served as an inoculum for a third tube (1:1000 dilution of the original inoculum). All tubes were incubated in the dark at 16°C, and cultures were maintained in sterilized 0.2-μm filtered lake water by replacing 1 ml of culture with 1 ml of *P. versatilis* culture every 7 to 11 days. Flagellate growth was assessed by epifluorescence microscopy (see Supplementary Information). From tubes showing heterotrophic flagellate growth, those with the highest dilution were further subjected to a dilution-to-extinction approach. In case of no growth in the 1:1000 dilution, the corresponding 1:100 dilution was used. Samples were serially diluted to adjust the abundance of HNF to 1 cell per well in a 24-deep-well plate containing 9 ml of sterilized 0.2-μm filtered reservoir water as medium and *P. versatilis* as prey. The plates were sealed with silicon covers, incubated in the dark at 16°C, and fed with prey every 7–11 days. Individual wells were screened microscopically for growth by staining of a subsample with DAPI as described above. Wells with no cells were discarded.

A total of 656 enrichment cultures were screened using CARD-FISH with probe Cry1-652. Pooled aliquots from enrichment cultures were hybridized with the CARD-FISH probe in multiple batches. If a positive signal was obtained, then the pooled aliquots were separated into two halves and hybridized again. Successive examination of the positive fraction was continued by halving the pooled cultures until the exact sample with the CARD-FISH–positive culture was identified. Out of the 656 enrichment cultures, we identified two cultures with CRY1a-positive signals. PCR amplification of the 18S rRNA gene was performed for both cultures using EukA and EukB primers [25]. Sanger sequencing confirmed one culture to be pure CRY1a, whereas the other was a mix of CRY1a and a *Spumella*-like chrysophyte. The pure culture was maintained in the dark at 16°C and fed with *P. versatilis* every 7–11 days. All results in this work are based upon the pure culture that was propagated for further genomic investigations. For details on culture propagation, growth curves, DNA/RNA isolation, electron microscopy, transcriptome sequencing, and assembly, see Supplementary Information.

### Genome assembly, gene prediction, and annotation

Briefly, isolated DNA was sequenced by long-read (Oxford Nanopore) and short-read (Illumina) technologies. Long reads were assembled using Flye v2.9.1 [26] and Canu v2.2 [27], prokaryotic contigs were removed, and long reads were extracted after re-mapping to combined assemblies. These long reads were re-assembled with Canu v.2.2, and any remaining prokaryotic contigs were also removed. A mitochondrial genome of 48 kb was recovered from this assembly. The decontaminated draft genome was polished in three rounds using Illumina reads with Pilon v1.24 [28]. Genome completeness was estimated using BUSCO v5.5 [29] using the conserved eukaryotic ortholog set (eukaryota_odb10, *n* = 255). Extended details on assembly, genome polishing, telomere identification, gene prediction, repeat analysis, and cryptomonad phylogenomics are provided in Supplementary Information.

### Endogenous viral element identification and analysis

Polinton-like viruses and virophages in the CRY1a genome were identified using hallmark major capsid proteins (MCPs), shifts in GC content, and the presence of characteristic flanking repeats. Candidate loci were curated in Geneious v9.1.3 (https://www.geneious.com) where dot plots were used to detect terminal repeats—either terminal direct repeats (TDRs) or terminal inverted repeats (TIRs). Flanking sequences (20 bp upstream and downstream) of each element were manually inspected for the presence of target site duplications (TSDs) (Supplementary Table S2). Protein sequences were clustered into orthologous groups using MMseqs2 (easy-cluster mode) [30] and annotated using InterProScan [31], TIGRFAMs [32], COGs [33], and KOfamKOALA [34]. MCP sequences representing all known PLV groups and relatives, along with all those recovered from *T. regina* PLVs (*n* = 21), were collected (total *n* = 1376), aligned using MAFFT (E-INS-i mode), and a maximum-likelihood tree was made using IQ-TREE2 (-m TEST --perturb 0.2 --nstop 500 -B 1000 --alrt 1000) with the best-fitting model (Q.pfam+F + G) selected according to Bayesian Information Criterion [35, 36]. Resulting phylogenetic clusters were annotated according to labelled MCP references. PLV annotations and orthologous group (OG) assignments are available in Supplementary Table S3. Additional details regarding PLV recovery, annotation, and MCP phylogeny are included in Supplementary Information.

### Giant virus genome recovery, phylogenomics, and metagenomics

In the long-read assembly, a single giant virus belonging to the phylum *Nucleocytoviricota* was detected using genomad [37], but the genome could not be completely resolved because of extremely long TIRs of >100 kb. A second round of sequencing was done on the viral fraction (details in Supplementary Methods). The genome was re-assembled using Canu v2.2 and aided by repeat resolution using Elloreas [38]. Gene predictions in these genomes and reference giant viral genomes were performed using Prodigal v2.6.3 in the metagenomic mode [39], and seven conserved proteins of giant viruses were identified using HMM searches [40]. These were aligned and concatenated, and a maximum-likelihood tree was inferred using IQ-TREE2. Additionally, assembled contigs from multiple freshwater metagenomes [41–46] were compared to viral genomes to identify similar contigs retrieved from previous studies. Viral abundance (RPKM, reads per kilobase per million mapped reads) in short-read metagenomic datasets was estimated using CoverM [47]. Genomes with >0.5 covered fraction were considered present; RPKM was otherwise set to zero (see Supplementary Table S4). Detailed methods for the assembly of viral genomes, phylogenomics, and metagenomics analyses are provided in Supplementary Information.

## Results and discussion

### Global biogeography

**Figure 1:**
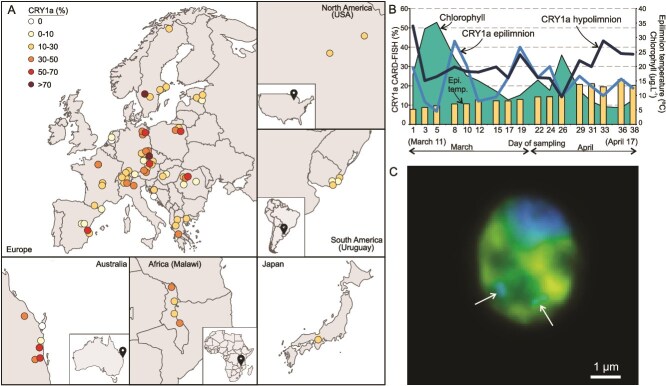
CARD-FISH analyses of the CRY1a lineage. (A) Geographic locations of all samples used for CARD-FISH in this work. Samples are categorized according to the maximum of CRY1a (%) to total HNF found at the site (key top left). (B) Percentage of CRY1a using CARD-FISH in epilimnion and hypolimnion in the Řimov Reservoir during the spring phytoplankton bloom (2024). Chlorophyll concentrations and epilimnion temperatures are shown on the secondary axis. Start–end dates of sampling are indicated at the ends of the *x*-axis. (C) Microphotograph created using an overlay of Z-stack images showing typical CRY1a morphology with ingested prey (indicated by white arrows). CRY1a is targeted by a specific CARD-FISH probe and FITC stained in yellow. DAPI-stained bacteria and CRY1a nucleus are coloured blue. A scale bar of 1 μm is shown at bottom right.

We examined 263 CARD-FISH filters hybridized with a lineage-specific probe from 84 lakes/reservoirs and 3 lagoons to determine the geographic distribution and abundance of the freshwater CRY1a lineage (Fig. 1). All observed counts for CRY1a and HNF, along with physicochemical parameters for each sample, are provided in Supplementary Table S1. In more than half the samples (*n* = 154), CRY1a represented >10% of all HNF, and in only 39 samples (14%), its abundance was <1%. CRY1a was found to be abundant in samples from all five continents, as well as in the southern hemisphere, revealing its ubiquitous distribution (Supplementary Table S1). Averaged across all samples, CRY1a accounted for ca. 17% of the HNF community, with generally high abundances across a variety of samples (*n* = 263, mean 16.47%, median 12.08%, SD 15.95%, range 0%–96.5%). We did not observe any strong correlations (*r* > 0.5*)* with any physicochemical parameter (Supplementary Table S5), suggesting that CRY1a is a remarkably versatile lineage with the ability to withstand a wide range of environmental gradients, except for very acidic lakes, where it was usually not found (Supplementary Table S1). We also sampled a high-resolution time series during a spring phytoplankton bloom in the Řimov Reservoir (April–May 2024), sampling both the surface (0.5 m) and hypolimnion (30 m) for 17 sampling days (over 38 days). CRY1a abundances in the epilimnion showed dramatic fluctuations throughout the sampling period (Fig. 1B), twice reaching maxima of ca. 40% of the HNF population. Abundances in the hypolimnion appeared high initially, then stabilized and started to increase towards the end of the bloom. However, at all times the populations remained relatively high (ca. 20%–30% of all HNF), indicating significant bacterivory by CRY1a, as also shown before [3]. Similar trends have also been described earlier at the same site [41]. The fastest doubling time of CRY1a in the Řimov Reservoir was 26.8 h, within the range of 24–32 h as estimated from hypertrophic shallow ponds [48].

### Isolation of CRY1a: *Tyrannomonas regina*

We implemented a dilution-to-extinction protocol (ca. 1 cell per well) coupled with genome-streamlined *Planktophila versatilis* as prey for isolating tiny heterotrophic flagellates (see methods). *Planktophila* spp. are small and extremely abundant in all freshwater habitats, including our sampling site, the Řimov Reservoir [21, 41, 42], and are a likely prey for a small and abundant heterotrophic flagellate. Upon screening with the specific CARD-FISH probe for CRY1a (see Methods), we identified a pure CRY1a culture, which was confirmed by 18S rRNA gene sequencing and propagated using *P. versatilis* prey bacteria (see Methods). We propose the name “*Tyrannomonas regina*” (i.e. Tyrant Queen, “*Tyrannos*,” Greek for “tyrant” or “ruler,” suffix “*-monas*,” Greek for “unit” or “single,” and “*regina*,” Latin for “queen”) for this isolate (*T. regina* strain RIE21), which is the first representative of the freshwater CRY1a lineage to be brought into culture.

**Figure 2:**
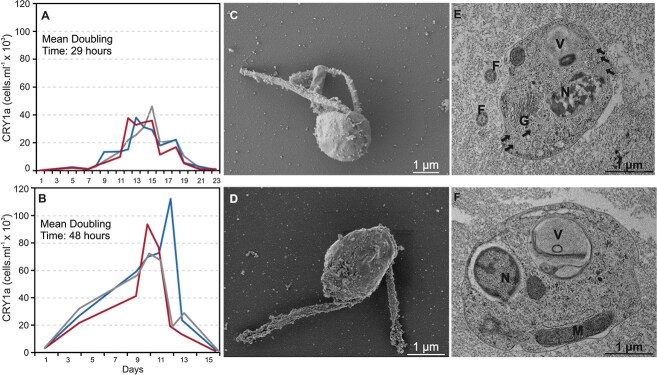
Growth curves and microscopy of *Tyrannomonas* culture. (A, B) Growth curves of *Tyrannomonas regina*. DAPI counts of cells are shown on the *y*-axis. A and B are separate experiments and each line represents an independent biological replicate. (C, D) Scanning electron micrographs of *T. regina* cells. (E, F) Transmission electron micrographs of *T. regina* cells. N, nucleus; M, mitochondrion; V, food vacuole with visible prey bacteria; F, flagella; G, Golgi apparatus. Black, solid arrows indicate the location of potential giant viruses in the cytoplasm. A scale bar of 1 μm is shown at bottom right for all electron micrographs.

Two independent experiments were performed to determine growth curves of *T. regina* using heat-killed *Polynucleobacter* bacteria in excess (see Methods) as prey, as it has been shown that CRY1a populations grow well with this microbe [3]. In the first (with an inoculum of 200 cells ml^−1^), the lag phase lasted ca. 1 week, and cultures grew rapidly up to a density of 40 000 cells ml^−1^, but two replicates showed a sudden collapse of at least half the population (Fig. 2A). The doubling time was 29 h, which is similar to prior *in situ* estimates (26.9 h in Řimov Reservoir and 24–32 h in hypertrophic ponds) (Supplementary Table S6) [48]. In the second experiment, which started with a higher inoculum (ca. 3500 cells ml^−1^), the lag phase was practically absent, and cultures reached maximal density of up to 110 000 cells ml^−1^ but at a much slower pace (doubling time 48 h) (Fig. 2B). In this case, the observed culture collapse was even more pronounced, with cell density dropping by nearly 80% in one replicate. The stationary phase, if any, was extremely short in both experiments.

Scanning electron microscopy (SEM) revealed slightly ovoid cells with length 2.0–3.9 μm (average 3.04 μm, SD = 0.34, *n* = 22) and width 1.8–3.9 μm (average 2.44 μm, SD = 0.39, *n* = 22). Two flagella of unequal length were observed. The length of the longer flagellum was 3.98–6.8 μm (average = 5.35 μm, SD = 0.76, *n* = 22) and that of the shorter flagellum was 1.76–3.72 μm (average = 2.73 μm, SD = 0.0.59, *n* = 22). The estimated cell volume was 3.43–30.7 μm^3^ (average 10.38 μm^3^, SD = 4.56, *n* = 22). Similar biovolume estimations have also been reported from natural populations targeted by lineage-specific CARD-FISH probes (average 18.4 μm^3^, SD = 9.2, *n* = 105) [48] and are also concordant with those obtained from DAPI-stained cells from culture (see Taxonomic Summary below).

Intracellular structures were visualized using transmission electron microscopy (TEM), and the nucleus, mitochondrion, Golgi apparatus, and food vacuole were discernible. No ultrastructural elements with multiple lipid bilayers corresponding to plastids or nucleomorphs were evident. Unexpectedly, multiple small round bodies with a dense core (size ca. 100 nm) were also visible in the TEM images (Fig. 2E, 2F and Supplementary Fig. S1). These electron-dense bodies are reminiscent of giant viruses infecting protists, as similar structures have been described before [49–51]. We did not observe any free viral particles in the EM preparations, but these observations led us to suspect that the culture was infected by a giant virus that was also responsible for the inconsistent growth and collapse of the *T. regina* culture in the growth curve experiments.

### A high-quality draft of the *Tyrannomonas regina* genome

Genome sequencing of the *T. regina* culture was performed using long- and short-read sequencing. A total of 10.7 Gb of sequenced data consisting of 1 M high-quality long reads (Q ≥ 10) was obtained for genomic DNA. As the culture also contained a variety of bacteria in addition to the added prey bacteria, we performed binning to segregate prokaryotic and eukaryotic genomic bins and the entire assembly was also screened for contigs of viral origin. We recovered 188 contigs originating from the nuclear genome of *T. regina* (total 68.96 Mb, GC% = 51.7%, minimum contig length = 10.8 kb, maximum contig length = 780 kb, average contig length = 366.8 kb, N50 = 350 kb, coverage = 30x). The assembled genome was estimated to be 78.8% complete, and a similar estimate of 80.8% was obtained from the assembled transcriptome [29]. However, these are likely to be under-estimates as they were obtained by using a conserved set for eukaryotes, there being no conserved gene sets available for evaluating genome completeness for cryptomonads yet.

Of the 188 contigs, 133 had recognizable telomeric repeat sequences at both ends, suggesting that they represented complete chromosomes recovered telomere-to-telomere (Supplementary Table S7). At least one high-quality telomere was identified in an additional 38 contigs. These indicate that the majority of the recovered genome was in complete chromosomes. The recovery of 133 telomere-to-telomere contigs and 38 contigs with at least one telomere suggests that there are at least 152 chromosomes in a single *Tyrannomonas* cell. We also recovered a complete circular-mapping mitochondrial genome (likely circular, length 48 kb). No nucleomorph or plastid genomes were recovered, suggesting the absence of any extant resident red-algal endosymbiont relicts in *T. regina*, but more detailed analyses of the nuclear genome will be necessary to fully confirm the complete absence of any genes from a past endosymbiotic event, as performed for the goniomonad *N. avonlea* [14]. Taken together, these results suggest that we retrieved a considerable fraction of the *T. regina* genome, allowing for robust genomic analyses and comparative genomics.

**Figure 3:**
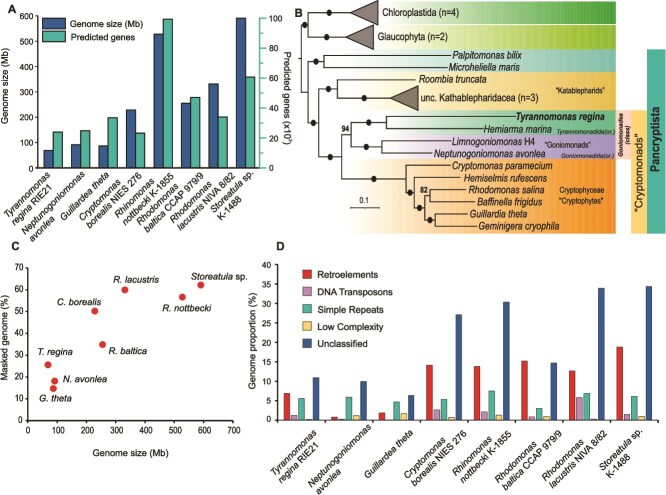
Genomic characteristics and phylogenomics of Cryptista. (A) Genome size and number of predicted genes in cryptomonad genomes. (B) Phylogenomic tree of Pancryptista (using transcriptomes and genomes, see Methods). Solid dots indicate 100% UFboot support. Chlorophytes and glaucophytes are used as the tree outgroup and the tree scale (estimated number of substitutions per site) is shown at bottom left. (C) Genome size in cryptomonads in relation to increase in masked genome content (repeats and low-complexity regions) in cryptomonad genomes (*y*-axis shows % of genome masked by RepeatMasker). (D) Repeat types in cryptomonad genomes as identified by RepeatMasker.

The assembled *T. regina* nuclear genome harbours 21 321 genes. Of these, 2598 appear to be alternatively spliced, with the maximum number of isoforms being eight. This is considerably lower than the ca. 33 000 genes predicted in the larger *N. avonlea* genome (92 Mb) [13, 14]. The number of predicted genes in the *T. regina* genome is also markedly lower than those in most genome sequenced cryptophytes (Fig. 3) [24]. Nine pairs of SSU and LSU rRNA genes were identified in the genome, in clusters of 7 and 2 in two separate contigs (Supplementary Table S8). The SSU rRNA gene was ≥99% identical in all these operons. The SSU rRNA gene tree suggests that the CRY1a lineage itself has several sub-lineages, with all *T. regina* sequences falling into one of these (see Supplementary Fig. S2). Twelve 5S rRNA genes were found but were not co-located with any of the SSU and LSU genes. Some appeared singly, and others in clusters (at least four clusters of two 5S rRNA genes each) (Supplementary Table S8). Transfer RNAs for 19 of the 20 proteinogenic amino acids were identified in the *T. regina* genome, with only tRNA-Asp missing (Supplementary Table S9). This may be due to extremely stringent filtering and retention of only contigs >10 kb in the final assembly. However, all tRNA synthetases, including those for asparagine, were identified (see below). All five major spliceosomal RNAs (U1, U2, U4, U5, and U6) were found. No minor spliceosomal RNAs were identified. All hallmark genes for phagocytosis were found. Several rhodopsins were identified (type-1), suggesting a photoheterotrophic or a sensory role (see details in Supplementary Information). No heliorhodopsins were found. Other metabolic characteristics of *T. regina* implied by the genome analysis are provided in Supplementary Information.

Phylogenomic analysis using 224 orthologous genes selected *via* PhyloFisher [52] placed *T. regina* closest to the heterotrophic cryptomonad *Hemiarma marina*, confirming the monophyly of the entire CRY1 clade (Fig. 3B, Supplementary Fig. S3). Together, they are a sister group to the clade formed by *Neptunogoniomonas* and *Limnogoniomonas* (formerly *Pseudogoniomonas*) [5, 13]. The cryptomonad clade comprising organisms with a known history of endosymbiosis is hereafter referred to as cryptophytes (Fig. 3). The *T. regina* genome is several fold smaller than most cryptophyte genomes (almost 10-fold smaller than the *Storeatula* sp*.* genome) owing to the genome expansions in the latter in which genome sizes are in hundreds of megabases (Fig. 3A). A substantial fraction of this increased genome size in cryptophytes is driven by repeat expansions, with more than half the genome constituted by repeat elements (ca. 60% in *Storeatula* and *Rhodomonas*) (Fig. 3B). Such extremely high percentages of genomic repeat content are not dissimilar to those observed in plant genomes. The *T. regina* genome, although smaller, shows higher repeat content than *N. avonlea.* However, this may be due to the use of short-read technology for the *N. avonlea* genome, which renders repeat recovery problematic.

**Figure 4:**
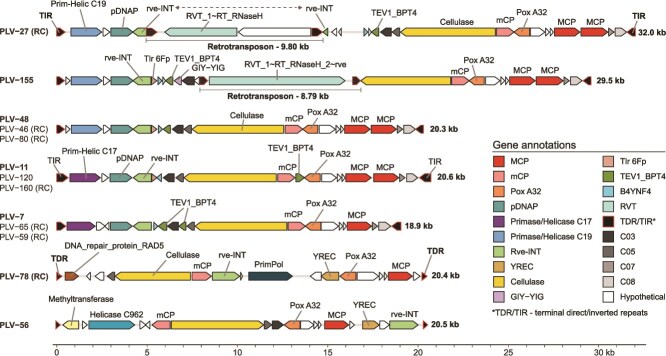
Representative polinton-like viruses (PLVs) from *Tyrannomonas regina*. Complete PLVs recovered from the *T. regina* genome are shown, each flanked by terminal inverted repeats (TIRs) or terminal direct repeats (TDRs), indicated by black arrowheads. Genes are distinguished by predicted function (see legend); hypothetical proteins are shaded grey and white. Conserved core genes involved in replication (pPolB, primase–helicase, etc.), integration (rve-INT, YREC), and morphogenesis (MCP, mCP, pox A32) are present in all PLVs. Retrotransposon-like insertions are underlined in the first two PLVs. Only one representative is shown for each group of nearly identical PLVs (names in bold), and all are oriented such that MCP genes are in the forward direction. Gene abbreviations: BANYNF4, V14_SPTNK uncharacterized protein present in sputnik virophage; RVT, reverse transcriptase; YREC, tyrosine recombinase; TEV1_BPT4, intron-associated endonuclease 1; pPolB, protein-primed DNA polymerase. Cluster membership is specified for frequently occurring genes without assignable function.

Post-acquisition of the red-algal endosymbiont, a pattern of genome expansion is discernible in cryptomonads. With the availability of genomes of goniomonadeans (*Tyrannomonas* and *Neptunogoniomonas*) and cryptophytes, it becomes possible to identify ancestral and specific repeat elements and their role in the evolution of cryptomonads. Overall, it seems that genome expansion in cryptophytes is mostly driven by retroelements and DNA transposons, and not only by simple repeats or low-complexity regions. For instance, elements related to Mavericks (DNA transposons also known as polintons) are found across all lineages, and whereas several other such transposons are found exclusively in cryptophyte genomes, their numbers do not appear to be greatly expanded (Supplementary Table S10). Linear interspersed nuclear elements (LINE) retroelements are found in all cryptophyte genomes but appear to be expanded in some, e.g. LINE/CRE in *Rhodomonas lacustris* and *Cryptomonas borealis* and LINE/Dualen in all cryptophytes (except *Guillardia theta*). Similarly, even though long terminal repeat (LTR) retroelements are common to all genomes, certain specific classes, such as LTR/Gypsy, show dramatic expansion in some cryptophytes, reaching 20–30 000 copies in *Cryptomonas* and *Storeatula* genomes (Supplementary Table S10). Another class of LTR retrotransposons is the Copia elements that are also expanded in cryptophytes, ranging from 4 to 24 000 copies in cryptophytes compared to <100 in goniomonadeans. However, the bulk of the repetitive regions in the genomes of cryptophytes, almost without exception, are as yet unclassified repeat elements (ca. 25%–35% of the total genome) (Fig. 4D).

### Polinton-like viruses in the *T. regina* genome

Recently, it has been shown that Polinton-like viruses (14–40 kb) are greatly expanded in cryptophyte genomes [24]. PLVs are double-stranded DNA (dsDNA) elements related to Maverick–Polinton transposons and giant virus parasitising virophages (~10 kb) frequently occur as endogenous viral elements in protist genomes [53, 54]. Polintons typically encode a hallmark protein-primed DNA polymerase (pPolB) and a retrovirus-like integrase (rve-INT); most also contain genes for a DNA-packaging ATPase (A32) and a maturation protease, and are flanked by terminal inverted repeats of several hundred base pairs with 6-bp target site duplications [55]. Initially regarded as DNA transposons, polintons were later shown to encode major and minor capsid proteins (MCP and mCP), indicating them as *bona fide* double-stranded DNA viruses [56, 57]. In contrast, most PLVs lack pPolB but encode one or more helicases, and often rely on tyrosine recombinases for integration [58, 59]. The co-occurrence of integrases and capsid proteins enable both integration and free-virus phases, and recent studies suggest that PLVs may also inhibit giant virus replication [60]. Scanning the *T. regina* nuclear genome for polinton, PLV, and virophage MCPs, and regions of elevated or reduced GC content led to the identification of 13 PLVs distributed across different chromosomes (Supplementary Table S2). This relatively low count likely reflects the comparatively smaller genome of *T. regina* (~69 Mb) in comparison to larger genomes of *Rhodomonas* and *Cryptomonas* (500–600 Mb) [24]. A similarly low number of PLVs (<10) is observed in the *N. avonlea* (91.5 Mb), although underrepresentation due to short-read sequencing limitations cannot be excluded.

*Tyrannomonas* PLVs had a median length of 20.29 kb and a GC content (35%) considerably lower than that of the host genome (51.7%). Most (*n* = 11) were flanked by TIRs (median 488 bp), whereas two (PLV-56 and PLV-78) presented TDRs (median 266 bp). The often-observed 5–6-bp TSDs at integration sites were present in nearly all PLVs encoding canonical rve-INT integrases, except PLV-120. However, even though TIRs are common, TDRs have rarely been reported in PLVs and only from metagenomic data, e.g. PLV-RED1 [58]. In *T. regina*, TDRs were found exclusively in PLVs encoding a tyrosine recombinase (YREC), indicating a distinct integration strategy. YREC is known to mediate site-specific recombination via a circular DNA intermediate [61], a process consistent with the presence of TDRs and one that typically does not generate TSDs upon integration [62]. TDR-flanked PLVs in *T. regina* also encode an rve-type integrase that is unrelated to the canonical rve-INTs found in TIR-flanked PLVs, resulting in the co-occurrence of two distinct integration enzymes. A similar case has been reported in virophages of *Cafeteria burkhardae*, particularly within mid-GC EMALEs [63]. The two YREC-encoding elements of *T. regina* also employ replicases consisting of primase–helicase fusion proteins, whereas all other PLVs found in the genome use the canonical (Polinton–Maverick) pPolB polymerase. Additionally, five PLVs encode DNA-unwinding helicases similar to those found in Mavirus (Supplementary Table S3).

To position the MCPs from *T. regina* PLVs in a phylogenetic context, a maximum-likelihood tree was constructed by including MCP proteins predicted from these PLVs, along with curated representatives from previously described groups and newly recovered sequences from six cryptophytes [24] (see Methods; Supplementary Fig. S4). All MCPs from *T. regina* clustered into a well-supported monophyletic clade, hereafter designated as the CRY1-PLV group. This clade is a sister group to the previously defined GKS2 cluster, which includes metagenomically recovered sequences [64]. Within the CRY1-PLV group, MCPs from YREC-encoding PLV-78 and PLV-56 with TDRs occupy basal positions, suggesting that they represent ancestral forms. Interestingly, eight PLVs contain duplicated MCP genes with conserved local synteny (see Supplementary Fig. S5). In contrast, three other PLVs with canonical rve-type integrases retain a single MCP copy and form a well-supported subcluster that shares gene content with the duplicated forms (see Supplementary Fig. S5). Overall, MCP phylogeny suggests that CRY1-PLVs are not closely related with those of cryptophytes, appearing closer to rhizarian MCPs comprising the PaM2 group. Additionally, conserved minor capsid proteins (mCPs) present in all recovered PLVs appear highly divergent from reference sequences available in annotation databases but show strong similarity to those found in PLVs of the rhizarian *Paulinella micropora*, matching a custom profile HMM built using previously confirmed mCPs from PLVs found within its nuclear genome (see Methods).

Aside from the conserved capsid proteins and DNA-packaging ATPase (Pox A32), all 13 PLVs identified in *T. regina* encode a large protein with features characteristic of fibre-like viral structural proteins. These proteins are long (average ~1500 amino acids) and are predicted to form obligate multimers with beta-propeller-like folds. Many show regions of high similarity to glycosyl hydrolase superfamily domains, which include sialidases and cellulases. Remarkably, the sialidase/cellulase-like domain is conserved across all 13 PLVs, despite considerable variability in overall gene content. This conservation suggests a shared functional role, possibly related to host recognition or attachment, and points to a high degree of host specificity among these PLVs.

Nested insertions of mobile genetic elements were identified in two PLVs (PLV-27 and PLV-155). In PLV-27, the rve-INT gene is disrupted by a 9806-bp retrotransposon flanked by 595-bp direct repeats. PLV-155 harbours an 8795-bp retrotransposon insertion with 369-bp direct repeats, but without apparent disruption of PLV genes. This retrotransposon also showed evidence of transcription activity, but no expression was detected for PLV-155 genes (see Supplementary Fig. S6). No additional copies of this retrotransposon were found in the genome, indicating that it is a unique and potentially active element. Its insertion without gene disruption suggests that PLV-155 may serve as a shuttle, enabling the horizontal spread of this retrotransposon. In contrast, the degraded retrotransposon in PLV-27 is transcriptionally inactive, and a nearly identical copy is found outside of PLVs on contig C8. Since it disrupts the essential rve-INT gene, PLV-27 is more likely a target of insertion rather than a vehicle for retrotransposon mobilization. Screening for non-coding RNA genes revealed no detectable candidates within the recovered PLVs.

### Giant viruses infecting *T. regina*

**Figure 5:**
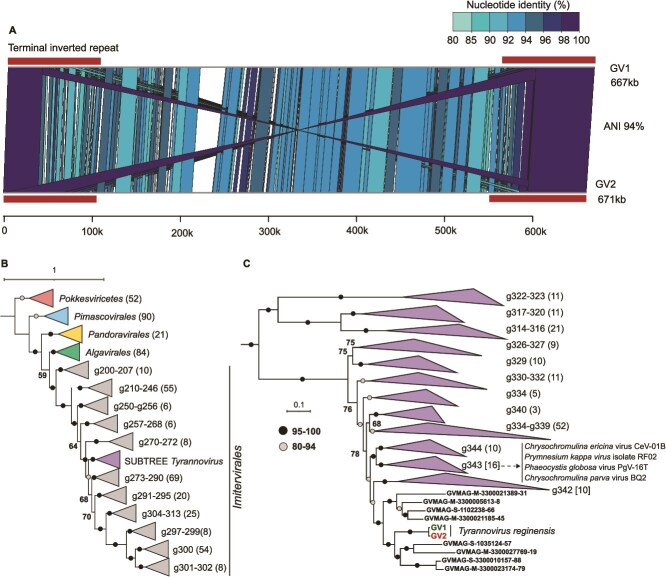
Genomic comparisons and phylogenomics of *Tyrannovirus* genomes. (A) Whole-genome comparison of the two giant virus genomes. Sequence identity blocks are coloured by % nucleotide identity (colour scale top right). The extents of terminal inverted repeats (TIRs) are indicated by a box. A length scale is shown at the bottom. (B) Phylogenomic tree of giant viruses showing relationships between giant viral groups. Subclades comprising multiple genera within *Imitervirales* are shown. (C) Close-up view of proposed giant virus genus *Tyrannovirus* (previously genus g341) in relation to other closely related giant viral genera. Closest isolate viral genomes (in genus g343) are indicated. Bootstrap values are shown at nodes in both trees.

We recovered two complete giant viral genome sequences (671 and 667 kb in length) from the *T. regina* genome assembly (Supplementary Table S11). These genomes were very similar to each other (average nucleotide identity 94%) and displayed characteristic features of genomes of nucleocytoplasmic large DNA viruses (NCLDVs). Both had long TIRs (ca. 100 kb), which are the longest described in any giant virus genome described before (Fig. 5). These TIRs also had similar but slightly different terminal hairpin sequences at both ends. Although it appears unusual that such highly similar viral genomes were assembled from the same culture, we found at least 43 proteins in each that had no detectable similarity to the other viral genome. Moreover, none of these singleton proteins showed any high sequence identity to the proteins encoded by the *T. regina* genome or any prokaryotic contigs from the assembly. Mapping of transcriptome-derived short reads against either viral genome did not yield any conclusive evidence of an ongoing infection process at the time of sampling of the transcriptome. We also did not observe any evidence of contamination by another protist (either in microscopy or in the assembled genome sequence). The recovery of these complete giant viral genomes, even after several months of culture propagation, coupled with the periodic catastrophic collapse, suggests that the culture was indeed infected concurrently by two similar but distinct viruses.

A phylogenomic tree using concatenated hallmark genes alignments placed both viruses within the order *Imitervirales* (Fig. 5). These genomes are the only isolated representatives of the genus “g341,” which only contains metagenome-derived giant viral genomes, the largest being 556 kb, recovered in eight contigs (GVMAG-S-1035124-57). After the host of these viruses, “*Tyrannomonas regina*,” we propose the name “*Tyrannovirus reginensis*” for these viruses (“*Tyrannos*,” Greek for tyrant or ruler, -virus, suffix for virus genera, and “*reginensis*,” Latin for “pertaining to” or “belonging to the queen/regina”). We will use the suffix GV1 and GV2 for these giant viruses.

*Tyrannovirus*-like contigs were recovered from a multitude of freshwater metagenome assemblies from geographically distant locations (Europe, Canada, and Australia), suggesting that viruses of this genus, similar to their host *Tyrannomonas*, are widely distributed across the world. However, most were short and only partially mapped to the genomes of *Tyrannovirus* isolates. The longest continuously aligned region consists of ~23 kb (contig LakePulse-ERR4869836-C926 of 25.3 kb) for both viruses. In the case of GV1, this region shows 1571 mismatches and 469 indels relative to the reference genome, whereas for GV2, there are 2185 mismatches and 568 indels. The small contig sizes, combined with observed sequence divergence, indicate a significantly higher genomic diversity than can be captured by isolate genomes alone. Moreover, the metagenomic contigs taken together did not map to the entire length of the isolated genomes, with several regions being completely unrepresented (Supplementary Fig. S7). This reiterates the need for obtaining viral isolates from cultured protists to capture complete genomes from major lineages of giant viruses.

Mapping of freshwater metagenomes to *Tyrannovirus* genomes also revealed high abundances of similar genomes across multiple continents (Supplementary Fig. S8, Supplementary Table S4). Moreover, in a metagenomic time series of the Řimov Reservoir, we could detect both viruses in the summer (mostly at the surface) (Supplementary Fig. S8). Fragment recruitment with samples of this time series revealed a large metagenomic island of ca. 27 kb (region 425–452 kb) (Supplementary Fig. S9). Remarkably, for GV2, practically the entire genome could be covered by the reads, and no large metagenomic islands were detectable (Supplementary Fig. S9), suggesting that near-identical viruses were present in this sample at this time. The metagenomic island detected in GV1 encoded methyl- and glycosyltransferases, enzyme classes that are implicated in modification of surface proteins (Supplementary Fig. S10). Similar proteins (especially with glycosyltransferase activity) are commonly observed in bacterial metagenomic islands [65], with different variants of the same metagenomic island offering a different “glycotype” for phage infection evasion [65, 66]. Phage genomes also show genomic islands of extremely high gene variability, particularly in regions that encode genes responsible for host recognition, e.g. tail fibres [67]. In protist-giant viral interactions, the actual genes that initiate strong contact are largely unknown, both for giant viruses and for the host. If, as is the case with bacteria and phages, such regions of high variability are critical for host–virus interactions [65], then it is entirely plausible that such genes will be important in host recognition. Moreover, it would be naturally expected that similar, highly diverse regions also exist in protist genomes and such genes encoded in these regions would be prime candidates for being the receptors for giant viruses.

### Concluding remarks

Protists are singularly underrepresented in genomic databases [68, 69], and this is especially true for environmentally abundant lineages. The use of 18S rRNA gene phylogenies has been instrumental in revealing hitherto unknown groups that are repeatedly recovered from multiple habitats. Traditionally, phototrophs and mixotrophs have been widely cultivated (e.g. *Rhodomonas*, *Cryptomonas*, *Chlamydomonas*, and *Ochromonas*-like chrysophytes among many others), but cultures of heterotrophic protists, even available ones, do not have similarly rich coverage. Moreover, and particularly in freshwaters, it was commonly believed that bacterivorous chrysophytes, like *Spumella*, were dominant as they were recovered frequently in bacterial prey-enriched cultures [22]. However, the importance of the feeding regime cannot be overstated once it became apparent that growth response of heterotrophic flagellates and the CRY1 lineage in particular was dependent upon prey “quality” [3]. Selective grazing by heterotrophs has been known for several decades and multiple studies have observed shifts in bacterial populations under grazing pressure [70–72]. We successfully exploited the “selective grazing” tendency of HNF, reasoning that one of the most abundant lineages of HNF would necessarily graze upon easily available *Planktophila*, representing abundant but tiny bacterioplankton members [21, 73]. In hindsight, this proved pivotal, as evidenced by the success in obtaining a pure culture of *Tyrannomonas*. This approach paves the way for isolation of other ecologically abundant bacterivorous flagellates that have so far resisted cultivation.

The genome of *T. regina* represents the smallest cryptomonad genome sequenced to date. It showed no evidence of any plastid or nucleomorph genomes, suggesting that, like *Neptunogoniomonas* [14], and consistent with their phylogenetic proximity, *Tyrannomonas* is also ancestrally aplastidic. However, further work is necessary to establish this conclusively, as there may be remnants of red alga–derived genes hidden within the nuclear genome. The *Tyrannomonas* genome also presents minimal repeat content in comparison to the substantial genome expansions observed in cryptophytes. This genome expansion in cryptophytes, attributable largely to LTR retroelements and other unclassified repeats, suggests that endosymbiosis conferred not just photosynthetic ability but also catalysed large-scale genome remodelling in the recipient. We have recently described multiple lineage-specific expansions of hundreds of copies of polinton-like viruses within cryptophytes [24], whereas only a handful (13) were found in the *T. regina* genome. Such endogenous viral elements represent not only historical encounters, but a growing body of evidence suggests they are also relevant for current infections [60, 74]. Fortuitously, the *T. regina* culture also yielded its own giant viruses that are likely responsible for the somewhat inconsistent growth of the pure culture. The two closely related giant viruses, *Tyrannovirus reginensis* GV1 and GV2, are the only isolates infecting a heterotrophic cryptomonad. Such giant viral genomes are critical towards deducing host–virus relationships from the vast amounts of metagenomic data that are currently available. Our successful cultivation and characterization of *T. regina* and its giant viruses sheds light on an enigmatic yet ubiquitous bacterivorous protist. It also offers a new path towards bringing into culture other important and abundant heterotrophic protists. Deciphering the biology of such previously inaccessible protist lineages will be critical towards understanding fundamental interactions in the aquatic microbial world.

### Taxonomic summary

Assignment of *Tyrannomonas regina* strain RIE21: Eukaryota, Cryptista, Cryptophyta/Cryptomonada, Goniomonadea (class), Tyrannomonadida (order), *Tyrannomonas* (genus), *Tyrannomonas regina* (species), strain RIE21.

#### Order Tyrannomonadida Mukherjee et al. 2025

##### Diagnosis

Monophyletic subgroup of Goniomonadea containing *Tyrannomonas regina* Mukherjee et al. 2025 and *Hemiarma marina* Shiratori but not *Goniomonas truncata* (Fresenius 1858).

##### Remarks

The order Tyrannomonadida presently has two described species, *Tyrannomonas regina* Mukherjee et al. 2025 and *Hemiarma marina* Shiratori and Ishida 2016, but environmental DNA sequences indicate the existence of a large diversity in two main clades that may be defined as two different families in the future. We define the order Tyrannomonadida as equal to the entire CRY1 clade, with the subclades CRY1a (including *T. regina*) and CRY1b (including *H. marina*) to be potentially formalized as two different families, pending further studies of their representatives.

#### *Tyrannomonas* Mukherjee et al. 2025

##### Diagnosis

A free-living, colourless, bi-flagellated, heterotrophic, and bacterivorous cryptomonad commonly found in freshwater habitats. Cells have ovoid shape and two flagella of unequal length. A furrow extends from the vestibulum for approximately half the length of the cell.

##### Etymology

The genus name is derived from “*Tyrannos*,” Greek for “tyrant” or “ruler,” with the suffix “*-monas*,” Greek for “unit” or “single.” It signifies *Tyrannomonas* as a prolific bacterivorous flagellate.

##### Type species

*T. regina* Mukherjee et al. 2025.

#### *T. regina* Mukherjee et al. 2025

##### Diagnosis

Free-living, colourless, bi-flagellated, heterotrophic, and bacterivorous cryptomonad found in diverse freshwater environments. Flagella of unequal length. Slightly ovoid cells with length 2.54–4.71 μm (average 3.64 μm, SD = 0.45, *n* = 52) and width 2.02–3.78 μm (average 3.02 μm, SD = 0.29, *n* = 52). Longer flagellum 4.2–7.5 μm (average = 5.59 μm, SD = 0.6, *n* = 52), shorter flagellum 1.9–4.1 μm (average = 2.81 μm, SD = 0.46, *n* = 33). Estimated cell volume 5–32.18 μm^3^ (average 18.05 μm^3^, SD = 5.13, *n* = 52).

##### Etymology

The species name “*regina*” is derived from Latin for “queen,” signifying the abundance and widespread distribution of *T. regina* in freshwater habitats.

##### Type location

The type strain (RIE21) was obtained from Řimov Reservoir, Czech Republic, 48.8475817N, 14.4902242E.

##### Type material

Resin-embedded cells of *T. regina* strain RIE21 were conserved at the National Museum of the Czech Republic in Prague. Deposition number: P6E 5656.

##### Gene sequence data

18S, 28S, and 5.8S rRNA gene sequences have been deposited in GenBank with the accession numbers OZ369539, OZ369540, and OZ369541 respectively.

##### ZooBank ID

urn:lsid:zoobank.org:act:7451C44D-350E-4D8F-852F-ECCC7B47CA2B.

## Supplementary Material

Supplementary_Figures_wraf271

Supplementary_Table_S1_CARD-FISH_data_wraf271

Supplementary_Table_S2_PLVs_CRY1_wraf271

Supplementary_Table_S3_PLV_gene_annotations_wraf271

Supplementary_Table_S4_Metagenomic_recruitment_coverM_GVs_wraf271

Supplementary_Table_S5_Correlation_Table_wraf271

Supplementary_Table_S6_Growth_Curve_Data_wraf271

Supplementary_Table_S7_Telomere_Identification_wraf271

Supplementary_Table_S8_CRY1_genome_annotation_wraf271

Supplementary_Table_S9_tRNA_genes_wraf271

Supplementary_Table_S10_Genomic_Repeats_Analysis_wraf271

Supplementary_Table_S11_Giant_virus_genome_annotations_wraf271

Supplementary_Table_S12_BUSCO_completeness_wraf271

Supplementary_Table_S13_Rhodopsins_wraf271

Supplementary_Information_wraf271

## Data Availability

All sequences, alignments, and phylogenetic trees generated in this work are available at Zenodo (http://dx.doi.org/10.5281/zenodo.15210452). The raw sequencing data generated in this study are publicly available in the European Nucleotide Archive (ENA) at EMBL-EBI under the project accession number PRJEB79909, including raw genomic and transcriptomic data, as well as assembled genomes of eukaryotes and viruses.
