# Supplementary material for: Cultivation, genomics, and giant viruses of a ubiquitous and heterotrophic freshwater cryptomonad
Source: ISME J. 2025 Dec 6;19(1):wraf271. doi: 10.1093/ismejo/wraf271 (PMC12747079; doi:10.1093/ismejo/wraf271)
Supplement: Supplementary_Figures_wraf271 [file supplementary_figures_wraf271.pdf]

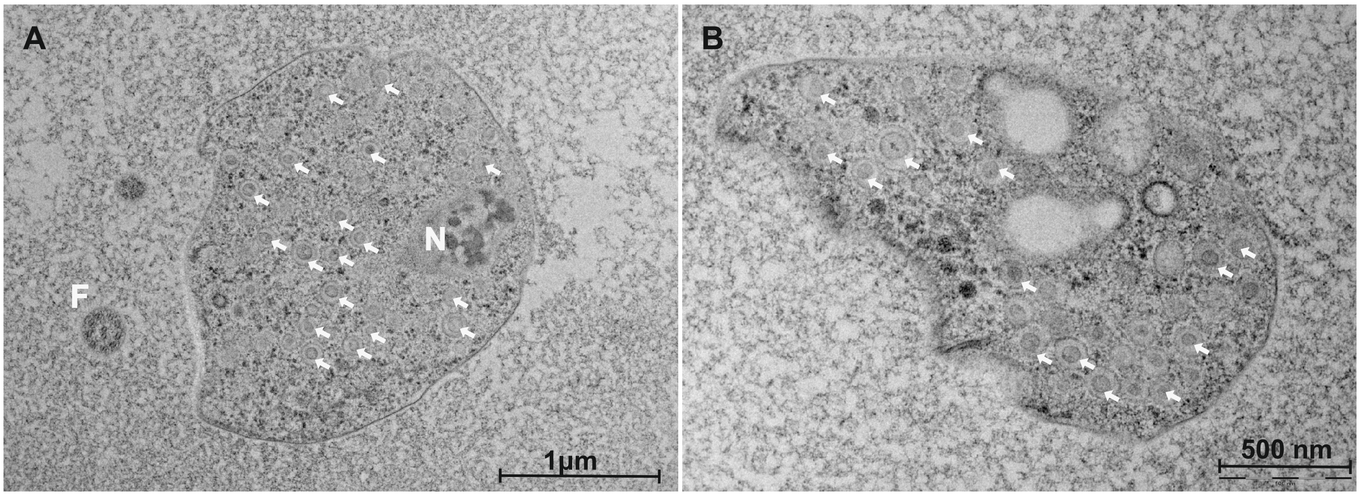

Supplementary Figure S1. A and B show transmission electron micrographs of *Tyrannomonas* cells. N-nucleus, F-flagella in cross section. Arrows indicate electron dense particles of roughly 100nm in size within the cytoplasm. Scale bars are shown at bottom right.

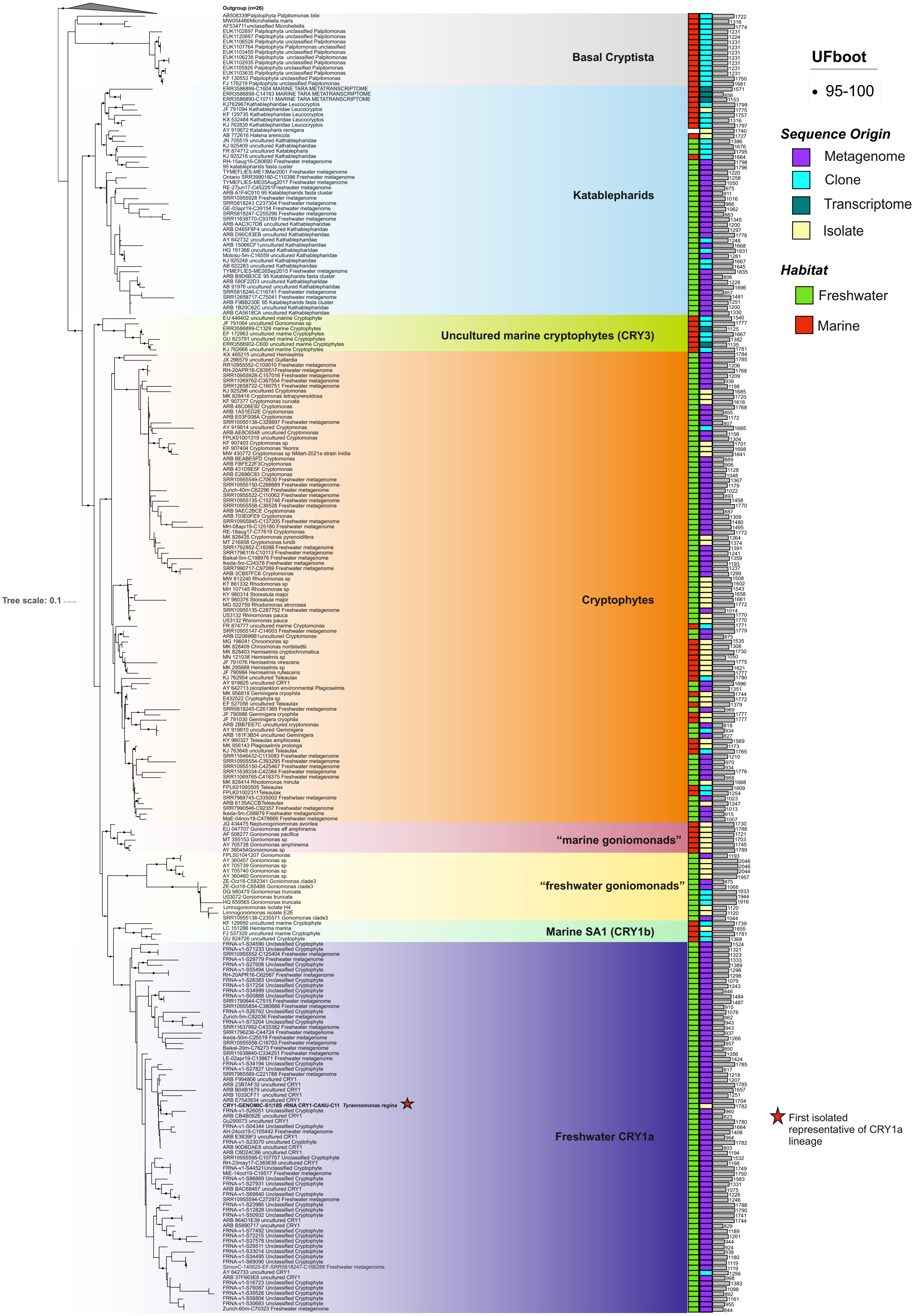

Supplementary Figure S2. Phylogenetic tree of Cryptista 18S rRNA gene sequences. Chlorophyta are used as outgroup taxa. Ultrafast bootstrap values are indicated in solid circles on the branches. Sequence origin and habitat are shown in colored boxes, legend is at top right.

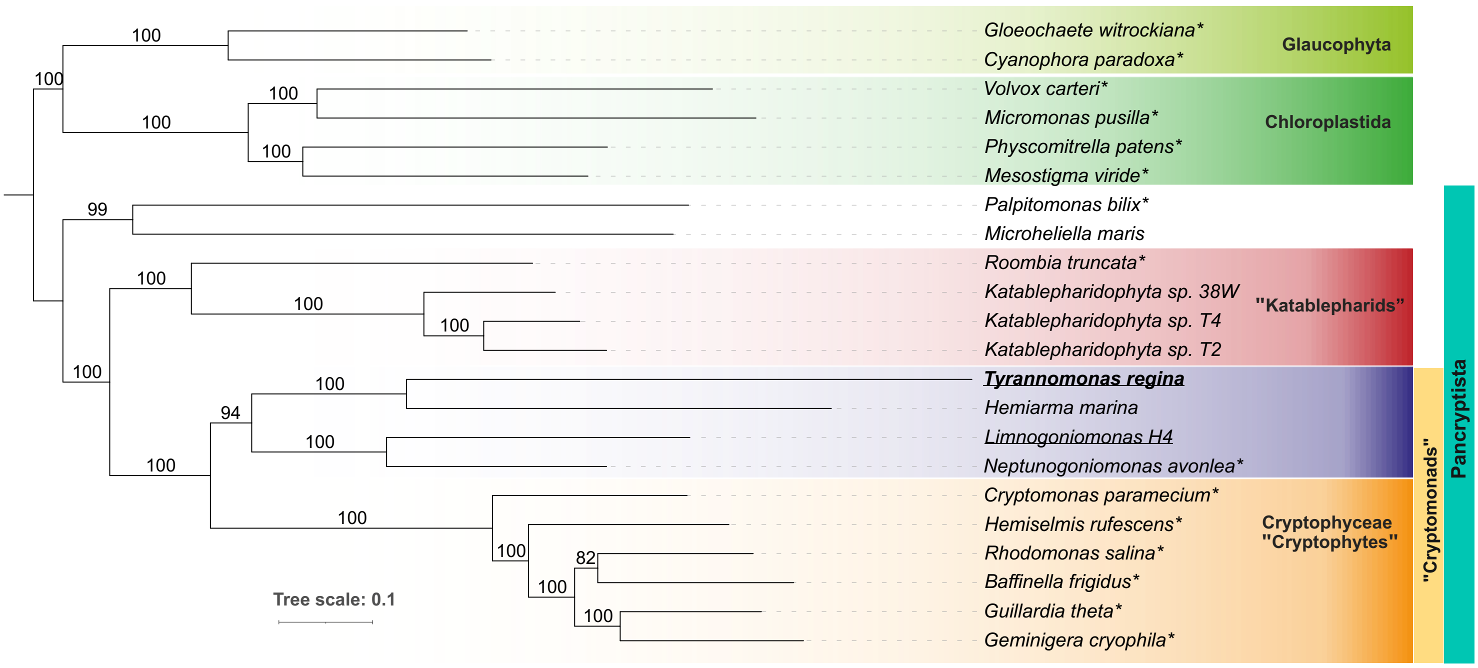

Supplementary Figure S3. Phylogenomic tree of Pancryptista. Ultrafast bootstrap values are indicated on the branches. Chlorophytes and glaucophytes are used as the tree outgroup and the tree scale (estimated number of substitutions per site) is shown at bottom left. An asterisk (*) indicates taxa already available in the PhyloFisher database. Taxa for which transcriptomes were generated in this work are underlined. Data for all remaining taxa was collected from public databases.

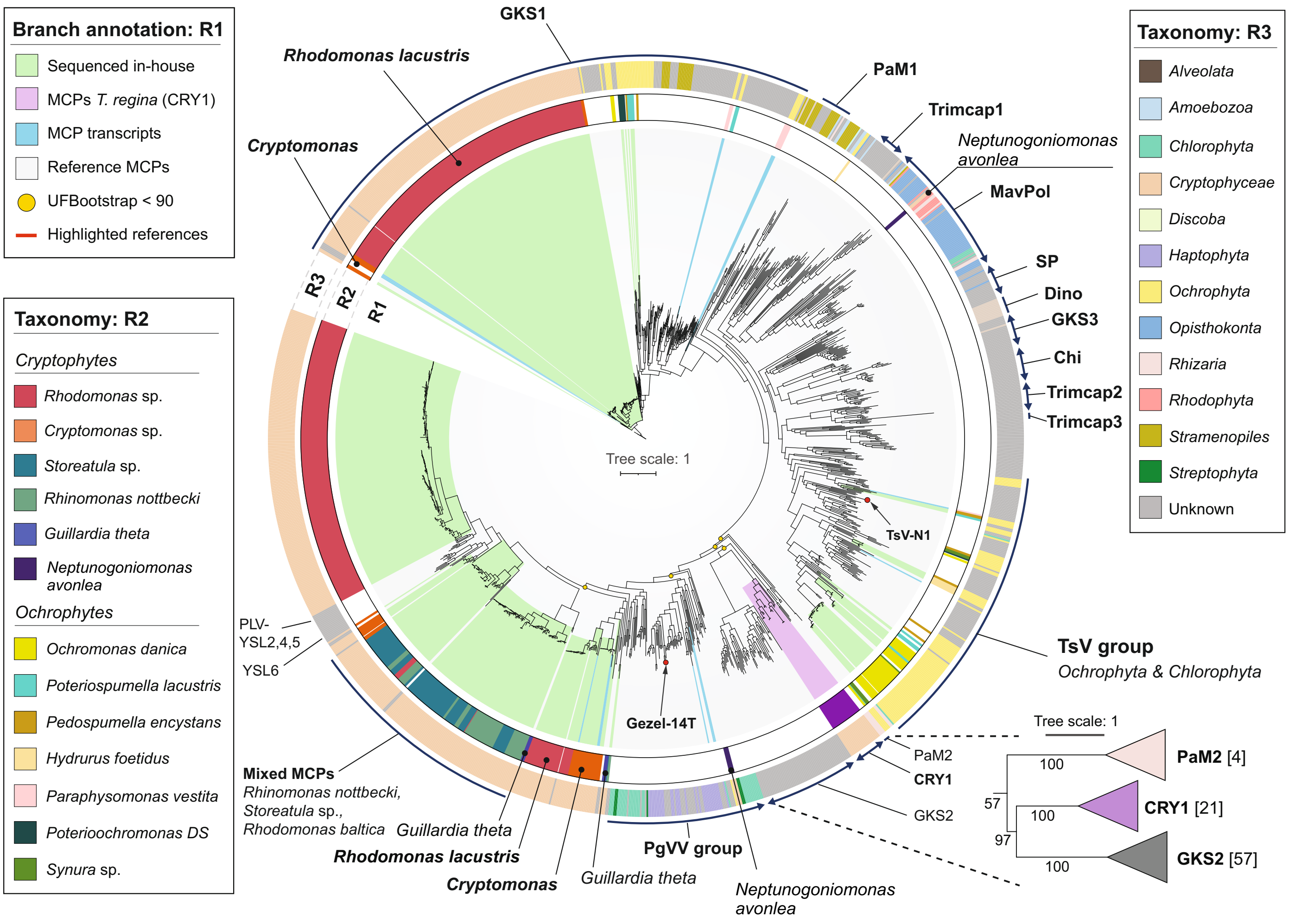

Supplementary Figure S4. Maximum-likelihood phylogeny of Major Capsid Proteins (MCPs) from Polinton-like viruses (PLVs). This unrooted tree shows complete MCP sequences from PLVs identified in this study and earlier work, including cryptophyte and ochrophyte PLVs, alongside reference sequences from genomic, metagenomic, and transcriptomic datasets. MCPs from *Tyrannomonas regina* PLVs form the distinct and well supported CRY1 cluster and are highlighted in purple (ring R1). A detailed view of this monophyletic cluster and neighboring clades GKS2 and PaM2 is shown at bottom right. Taxonomic annotations are shown in outer rings: R2: Genus-level taxonomy; R3: Major eukaryotic clades. Ultrafast bootstrap values (UFB) below 90% are indicated for relevant branches by yellow circles; others are ≥90%. Sequences derived from this study and previously defined reference PLV groups (e.g., PgVV, TsV, MavPol) are indicated.

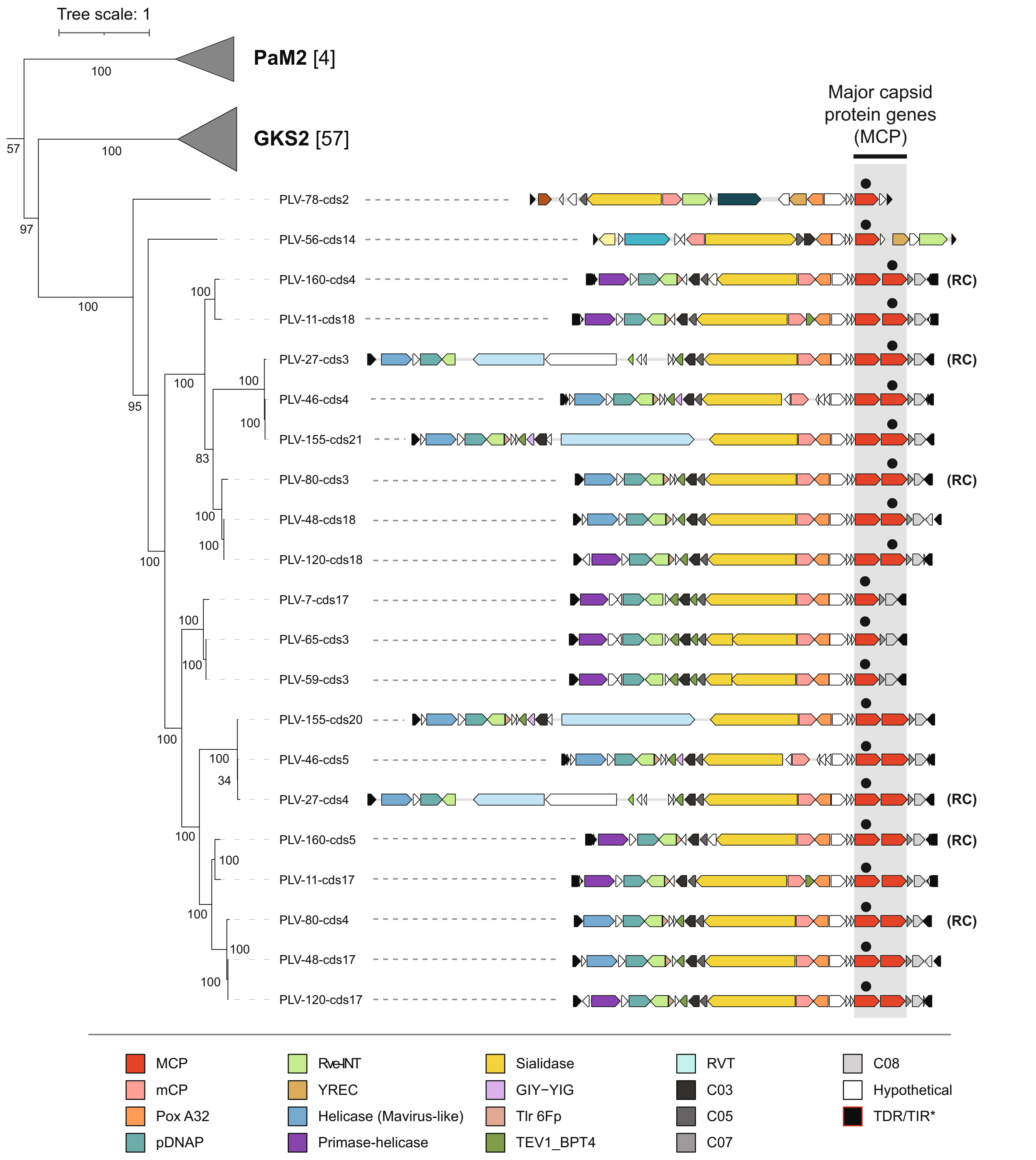

Supplementary Figure S5. Phylogenetic relationships and genomic context of duplicated MCP genes in Polinton-like viruses (PLVs). This figure presents a maximum likelihood phylogeny of Major Capsid Protein (MCP) sequences from PLVs, highlighting MCP duplications. MCPs from Tyrannomonas regina-associated PLVs form the CRY1 cluster. When a contig contains duplicated MCPs, both copies are included and marked with black circles; such contigs are shown twice to display both gene positions. Gene maps show the genomic context of each MCP, with genes color-coded by predicted function or marked as hypothetical. Reverse-complemented contigs are labeled “(RC)”. Neighboring MCP clusters (GKS2 and PaM2) are shown as collapsed nodes with the number of sequences in brackets. Branch support values are indicated. Abbreviations: TDR/TIR (terminal direct/inverted repeats) and RVT (reverse transcriptase) are noted where relevant.

PLV−155 - *Tyrannomonas regina*

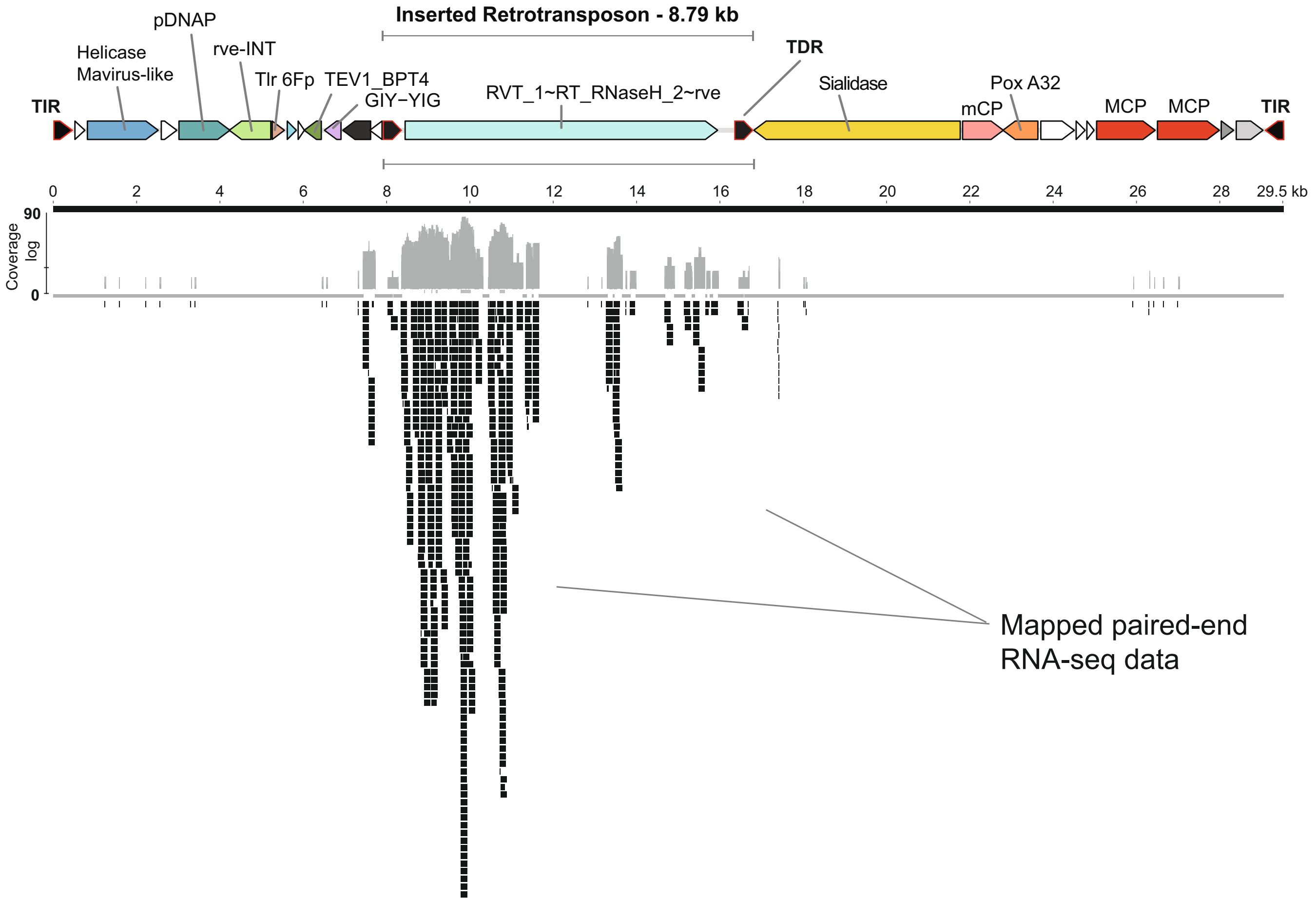

Supplementary Figure S6. Expression of a retrotransposon inserted in PLV-155 from *Tyrannomonas regina.* RNA-seq reads from a single time point were mapped to all PLVs, revealing expression only in a retrotransposon nested within PLV-155. The insertion spans ~8.8 kb and does not disrupt annotated core viral genes. Read coverage (log scale) is shown above, with individual mapped reads as black lines below. Abbreviations: TDR/TIR - terminal direct/inverted repeat.

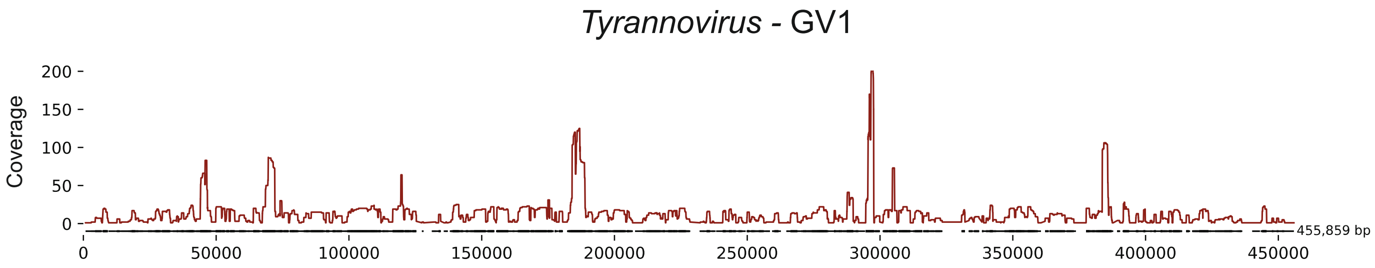

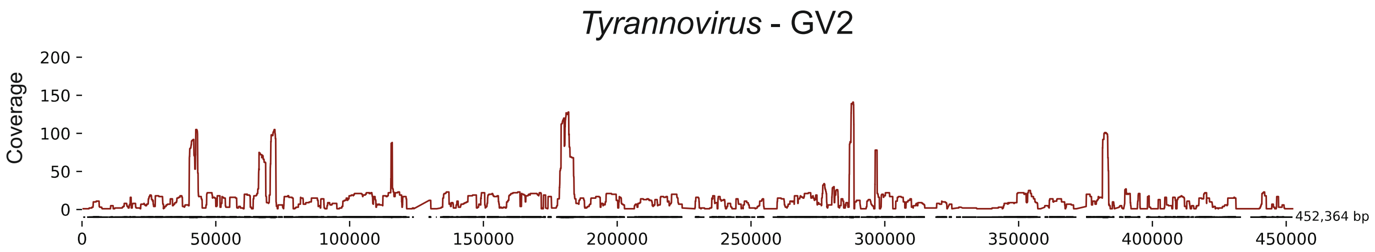

Supplementary Figure S7. Metagenomic contigs comparison to Tyrannoviruses GV1 and GV2. Assembled metagenomic contigs from diverse freshwater habitats representing multiple biomes were mapped to the core genomes (excluding terminal inverted repeat regions) of the two *Tyrannoviruses* infecting *Tyrranomonas regina*. Raw per-base coverage is shown across each genome, with contig alignment positions indicated below the x-axis. Coverage reflects unnormalized read depth.

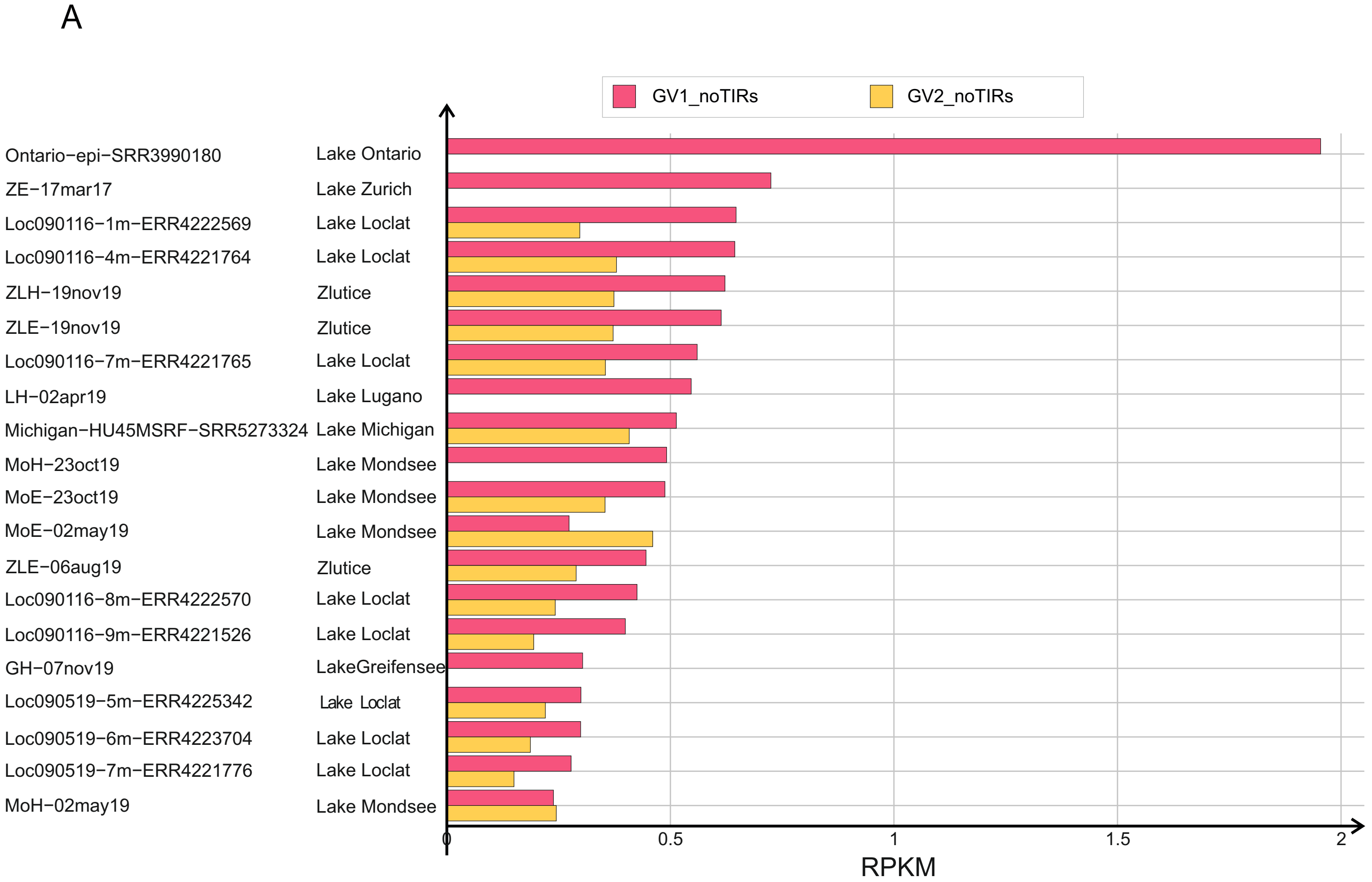

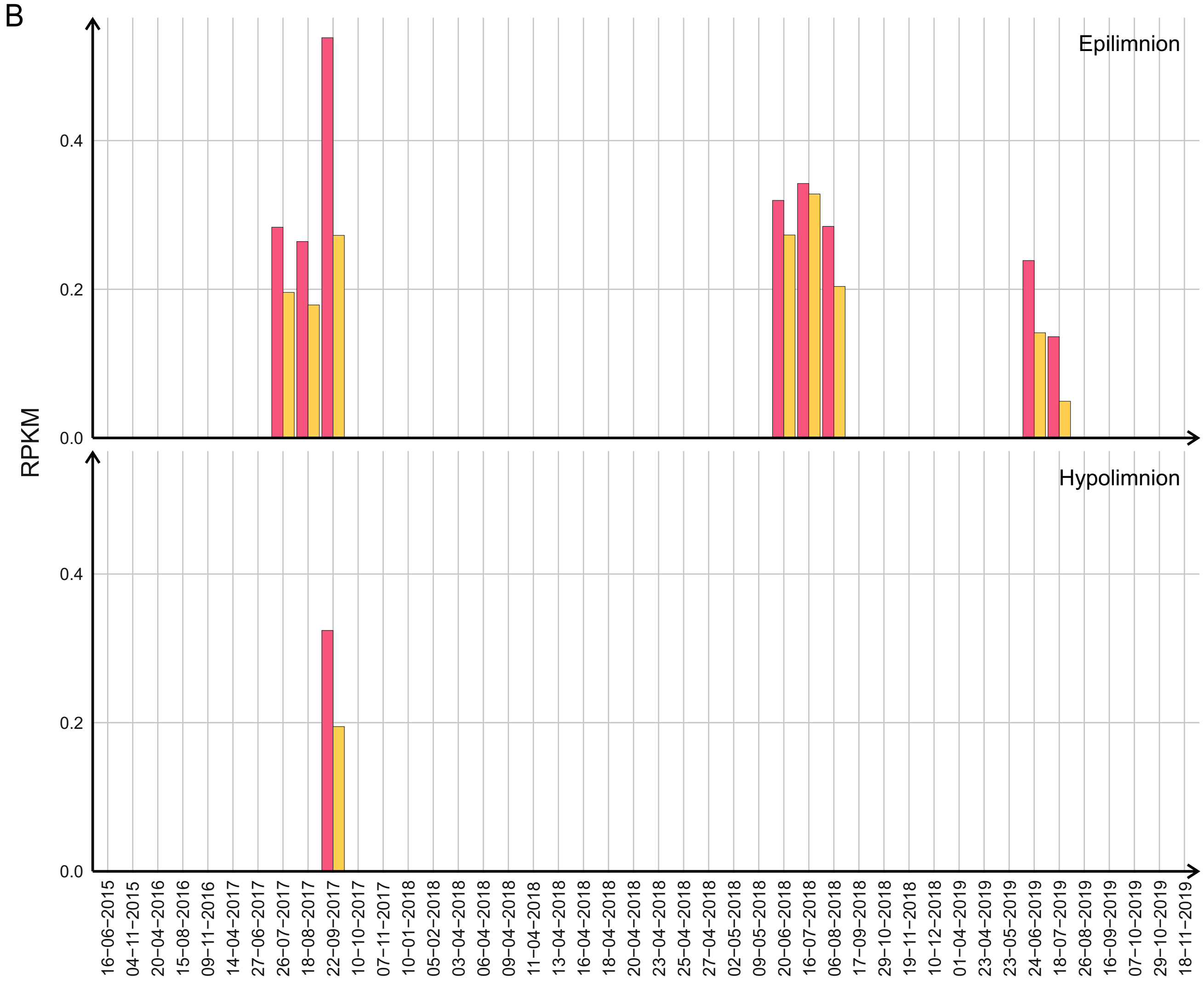

Supplementary Figure S8 (A) Metagenomic recruitment of the giant viruses GV1 and GV2 in publically available short-read metagenomes from freshwater lakes, the X-axis represents abundance expressed as RPKM (reads per kilobase million), and the Y-axis represents the metagenome. **(B)** Metagenomic recruitment of the giant viruses GV1 and GV2 in the Rimov reservoir metagenomic timeline. The X-axis represents a sampling date in the metagenomic timeline, and the Y-axis represents RPKM (reads per kilobase million)

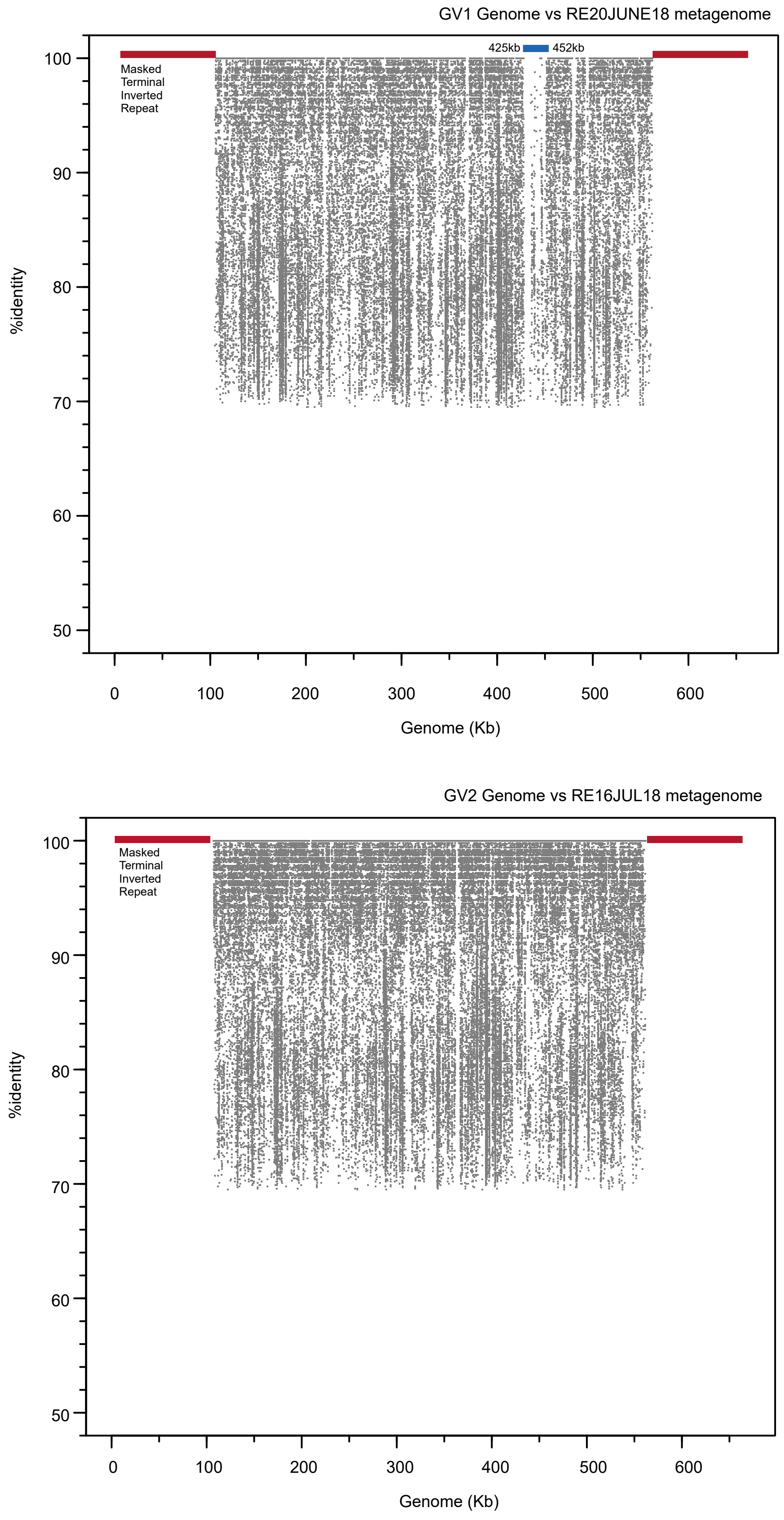

Supplementary Figure S9: Metagenomic fragment recruitment of GV1 (top) and GV2 (below) against two selected metagenomes from Rimov reservoir where these viruses are abundant. Masked terminal repeats are shown in red boxes and were not included in the sequence comparisons.Blue box in top figure indicates the location of the metagenomic island in the GV1 genome.

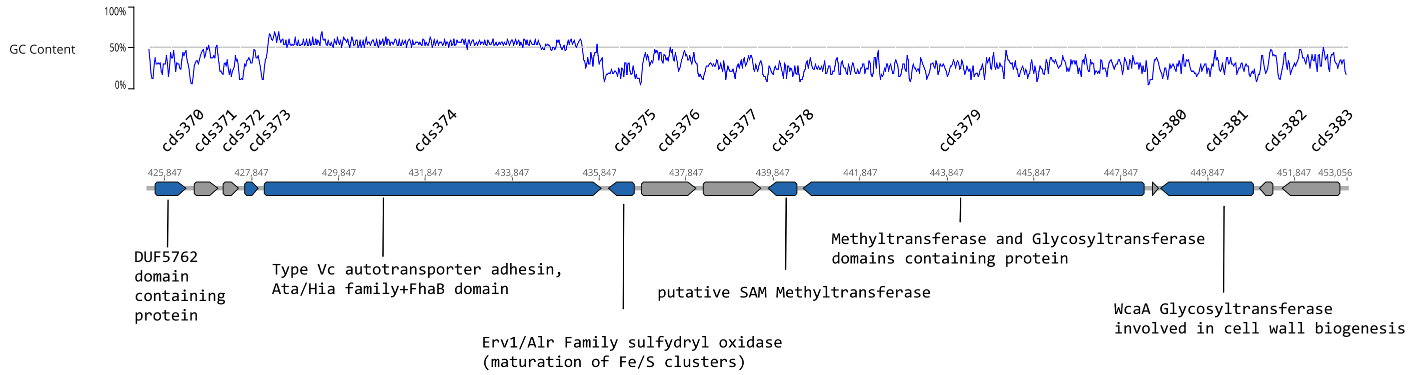

Supplementary Figure S10: Genes in the metagenomic island of *Tyrannovirus reginensis* GV1 giant virus. GC% plot is shown at the top, CDS features are numbered and locations in the genome are indicated above the genes. Gene annotations, whereever available, are provided. Hypothetical genes are colored in grey.

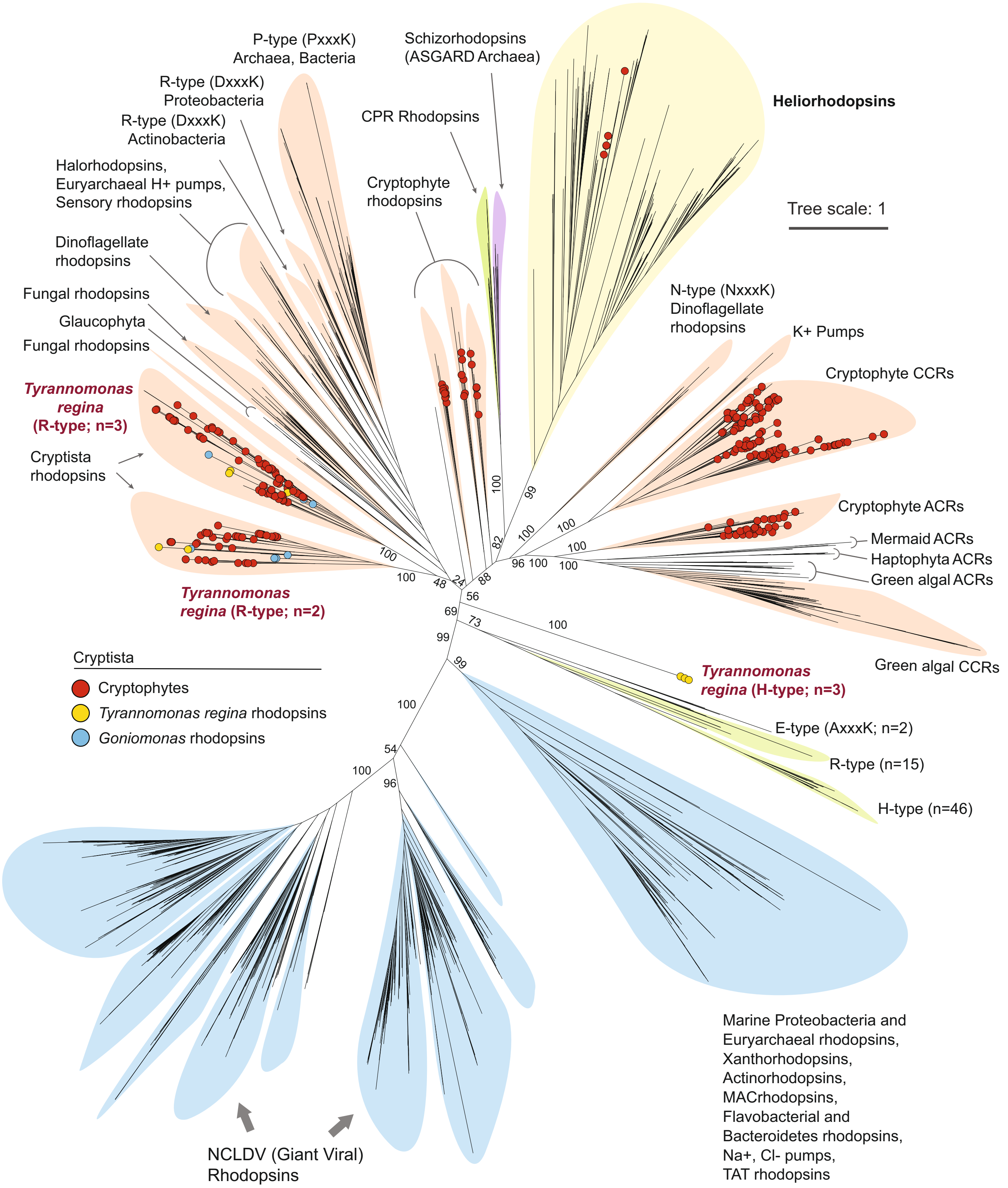

Supplementary Figure S11. Maximum likelihood phylogenetic tree of rhodopsins. Cryptista rhodopsins are marked with color-coded circles at the tips. Rhodopsins recovered from *Tyrannomonas regina* (n = 8) are highlighted with yellow circles; their type and count are indicated below the respective clades. Ultrafast bootstrap support values are shown at selected nodes.
