# Supplementary material for: Cultivation, genomics, and giant viruses of a ubiquitous and heterotrophic freshwater cryptomonad
Source: ISME J. 2025 Dec 6;19(1):wraf271. doi: 10.1093/ismejo/wraf271 (PMC12747079; doi:10.1093/ismejo/wraf271)
Supplement: Supplementary_Information_wraf271 [file supplementary_information_wraf271.pdf]

Supplementary Information

Cultivation, genomics, and giant viruses of a ubiquitous and heterotrophic freshwater cryptomonad

Indranil Mukherjee[1,†], Paul-Adrian Bulzu[1,†], Roudaina Boukheloua[1,2], Usman Asghar[1,2], Hongjae Park,[1,3], Helena Henriques Vieira[1], Maria-Cecilia Chiriac[1], Vojtěch Kasalický[1], Petr Znachor[1,2], Pavel Rychtecký[1], Karel Šimek[1], Michaela M. Salcher[1], Markus Haber[1], Rohit Ghai[1,*]

[1]*Biology Centre of the Czech Academy of Sciences, Institute of Hydrobiology, Na Sádkách 7, 37005, České Budějovice, Czech Republic*

[2]*Faculty of Science, University of South Bohemia, 37005, České Budějovice, Czech Republic*

[3]*Department of Biological Sciences and Bioengineering, Inha University, Incheon, Republic of Korea*

† Both authors contributed equally to this work

[*]*Corresponding author:* Rohit Ghai, email: ghai.rohit@gmail.com

Mailing Address: Laboratory of Microbial Ecology and Evolution, Department of Aquatic Microbial Ecology, Institute of Hydrobiology, Na Sádkách 7, 37005, České Budějovice, Czech Republic

Running Title: Cultivating an Elusive Cryptomonad

Keywords: cryptophytes, cryptomonads, flagellates, aquatic food web, freshwaters, biogeography, cultivation, CARD-FISH, whole-genome sequencing, giant virus

Table of Contents

Supplementary Materials and Methods 3

Enumeration of prokaryotes and protists. 3

CARD-FISH. 3

Growth curve of CRY1a. 3

Electron Microscopy. 4

DNA isolation and genomic sequencing. 4

RNA isolation. 5

Genome assembly, gene prediction and annotation. 5

Gene prediction, annotation and repeat analyses. 6

Transcriptome sequencing and assembly. 7

Phylogenomic tree of cryptophytes. 7

Recovery of giant virus genomes. 8

Phylogenomic tree of giant viruses. 9

Endogenous viral elements (EVE) identification and analysis. 9

Recovery of Tyrannovirus-like contigs from assembled metagenomes 12

Fragment recruitment analyses. 12

Phylogenetic analysis of small ribosomal rRNA sequences. 12

Phylogenetic tree of rhodopsins 13

Supplementary Results 13

Gene content and metabolic characteristics. 13

Cryptista rhodopsins. 14

Supplementary References 16

Supplementary Materials and Methods

Enumeration of prokaryotes and protists.

Water samples (50-100 ml) were immediately fixed after collection with filtered formaldehyde (2% final concentration). Duplicate samples were used to enumerate HNF (5–20 ml subsamples) on 1-µm pore-size filters (Osmonics, Livermore, CA). The samples were stained with DAPI (4',6-diamidino-2-phenylindole, 0.1 µg ml^{-1} final concentration), and HNF were counted via epifluorescence microscopy at 1000x magnification (Olympus BX53; Optical, Tokyo, Japan).

CARD-FISH.

Subsamples (10-50 ml) of formaldehyde fixed samples were filtered within one day of sampling onto white polycarbonate filters (0.8 µm pore-size, Sterlitech), and filters were stored at -20°C. CRY1a cells were targeted with the oligonucleotide probe Cry1-652 [1] purchased from Biomers.net (Ulm, Germany, PAGE-type purification). Details on quality and specificity of the FISH-probe are given in a recent review [2]. CARD-FISH was performed at 35°C for 2–3 h with fluorescein labelled tyramides following the protocol described before [2]. CARD-FISH preparations were analyzed by epifluorescence microscopy at 1000× magnification, and at least 50-100 HNF cells (wherever possible) were counted per sample (Supplementary Table S1). Images of probe positive cells were captured using an image analysis system (NISElements 5.1, Laboratory Imaging, Prague, Czech Republic).

Growth curve of CRY1a.

CRY1a cultures maintained with *Planktophila versatilis* were not fed for two weeks to reduce the background bacteria. Thereafter, the cultures were fed with heat-killed *Polynucleobacter* and acclimatised with feeding on this prey for one month in the dark at 16°C. *Polynucleobacter* was chosen over *Planktophila* as prey to boost the growth of CRY1a, as CRY1a was observed to grow faster and reach higher densities when fed with *Polynucleobacter [1]*. Cultures selected for measuring the growth curve were not fed with *Polynucleobacter* two weeks prior to the start of the experiment to minimise the presence of the previously added prey. At the start of the experiment, heat-killed *Polynucleobacter* was added to the CRY1a cultures 10 times higher than the bacteria already present in the culture in 500 ml flat-bottom borosilicate Erlenmeyer flasks. The experiments were run in triplicate in the dark at 16°C. Samples for CRY1a cell counts were collected daily and fixed with formaldehyde (2% final concentration) and stored at 4°C. Cells were enumerated on a 5 ml sample volume with DAPI staining under fluorescence microscope as mentioned above.

Experiments continued until the entire culture collapsed (details provided in Supplementary Table S6).

Electron Microscopy.

Transmission Electron Microscopy (TEM): The CRY1a culture was centrifuged at 500 × g and the pellet was high-pressure frozen using EM ICE (Leica) high-pressure freezer. Freeze substitution EM AFS2 (Leica) was carried out in 2% osmium tetroxide diluted in 100% acetone at -90 °C for 16 h, then warmed up at a rate of 5 °C per hour to remain at -20 °C for 14 h, and finally warmed up again at the same rate to a final temperature of 4 °C. Samples were rinsed three times in anhydrous acetone at room temperature and infiltrated stepwise in acetone mixed with SPI-pon resin (SPI) (acetone : SPI ratios of 2 : 1, 1 : 1, and 1 : 2, for 1 h at each step). The samples, now in pure resin, were polymerised at 60 °C for 48 h. Sections were prepared using an Ultracut UCT (Leica) microtome and collected on 300/400 mesh copper grids (SPI). Staining was performed using alcoholic uranyl acetate for 30 min and in lead citrate for 20 min. Images were obtained using a 1400 Flash (JEOL) transmission electron microscope.

Scanning Electron Microscopy (SEM): CRY1a cells were fixed in 2.5% (v/v) glutaraldehyde in 0.1 M phosphate buffer pH 7.2 and transferred to the poly-L-lysine coated coverslips, post fixed with 2% OsO_4 in 0.1 M phosphate buffer for 2 hours, dehydrated in ascending acetone series, critical point drying with CO_2 in Pelco CPD2 (Ted Pella Inc. Redding, CA, USA) and sputter coated with gold in a Sputter Coater Polaron chamber (Polaron Ltd., Watford, UK). The CRY1a cells were then observed in JEOL 7401-F (JEOL Europe, Prague, Czech Republic) at 4.0 kV accelerating voltage.

DNA isolation and genomic sequencing.

CRY1a cultures were grown in 1 L flat-bottom borosilicate Erlenmeyer flasks with 500 ml of 0.2 µm filtered and autoclaved freshwater collected from the Římov Reservoir. Cultures were supplemented with 5 ml of heat-killed *Polynucleobacter* and maintained in the dark at 16°C. Cells were harvested during the exponential growth phase by centrifugation at 3,000 × g for 10 minutes at room temperature. Resulting pellets were transferred into cryovials prefilled with 1 ml of DNA/RNA Shield Reagent (Zymo, R1200) and stored at −80 °C until nucleic acid extraction. For DNA isolation, preserved pellets were fully thawed at room temperature and lysed using Proteinase K (20 µl, 20 mg/ml; Zymo, D3001-2-20) and SDS (20 µl, 20% stock) for 30 minutes at 56 °C and 650 RPM on a thermoshaker (Grant Bio, PHMT-PSC24N). DNA was extracted using the Quick-DNA™ HMW MagBead Kit (Zymo, D6060), following the manufacturer's protocol for samples stored in DNA/RNA Shield. Elution from magnetic beads was performed with 200 µl of nuclease-free water. DNA concentration was measured

using a Qubit 3.0 Fluorometer (Invitrogen) with the Qubit dsDNA Broad Range Assay Kit (Thermo, Q32853). To meet sequencing input requirements (~100 ng/µl), eluted DNA was further concentrated using a SpeedVac (Thermo Scientific™, DNA120) for 20 minutes at 50 °C, then stored at −80 °C until shipment on dry ice.

Whole-genome shotgun Illumina sequencing (2 × 150 bp paired-end reads, 350 bp insert size) was performed at Novogene (Hong Kong, China), mainly to support polishing of the Nanopore long-read genome assembly. Short-read libraries were sequenced on the NovaSeq 6000 platform with a target yield of 20 Gbp.

Nanopore long-read sequencing was conducted in-house using a MinION™ Mk1B sequencing device (ONT). Libraries were prepared with the ligation sequencing kit V14 (SQK-LSK114; ONT) and run on a FLO-MIN114 (R10.4.1) flow cell for 72 hours.

RNA isolation.

Cultures were kept in light and dark conditions overnight before pooling for transcriptome sequencing with rRNA depletion to allow for improved coverage of transcripts for gene prediction. Cells stored in DNA/RNA Shield were lysed using Proteinase K and SDS, as described for DNA extraction, with incubation reduced to 15 minutes at 42 °C. A second lysis step was performed using TRI-Reagent (Zymo, R2050-1-200) at a 3:1 reagent-to-sample volume ratio for 15 minutes. RNA was extracted using the Direct-zol RNA Miniprep Kit (Zymo, R2050) following the manufacturer's protocol, with final elution performed twice using 25 µl of nuclease-free water preheated to 50 °C. RNA concentration was measured with a BioSpec-nano spectrophotometer (Shimadzu, CAT. No. 206-26300-38), and integrity was assessed by agarose gel electrophoresis. For quality control, ~4 µl of each sample was mixed with an equal volume of 2X RNA Gel Loading Dye (Thermo, R0641), heated at 70 °C for 10 minutes, and run on a 1% agarose gel in 1X TAE buffer. Samples meeting quality and quantity standards were stored at -80 °C prior to sequencing. RNA was isolated from the CRY1a culture obtained in this study, as well as from *Limnogoniomonas* (formerly *Pseudogoniomonas* H4), a heterotrophic cryptophyte related to *Neptunogoniomonas* that was previously isolated by us [3]. The transcriptome of *Limnogoniomonas* H4 was included in this work in order to enhance the resolution of the phylogenomic tree of cryptophytes.

Genome assembly, gene prediction and annotation.

Isolated DNA was sequenced by high-accuracy Illumina (Novaseq 6000) whole genome shotgun short-reads (PE150) and long-read Oxford Nanopore sequencing performed locally on a MinION machine (R10.4.1 flow cell chemistry). Raw Illumina reads were quality-filtered and adapter-trimmed using tools from the BBMAP suite (v38.86; https://sourceforge.net/projects/bbmap/). The Nanopore long reads were basecalled using

Guppy v6.5.7, and resulting FASTQ files were adapter- and barcode-trimmed using Porechop v0.2.4 [4]. Initial genome assemblies were generated from the long-read dataset to identify high-confidence eukaryotic contigs via taxonomy-based decontamination and coverage consistency. First, long reads were assembled independently using Flye v2.9.1 [5] and Canu v2.2 [6]. Assembled contigs were subjected to gene prediction using Prodigal v2.6.3 [7], and genes were taxonomically annotated. All contigs assigned to Prokaryota were removed, and the long-read dataset was mapped against the combined filtered assemblies (Flye and Canu) using minimap2 v2.18 [8]. Mapped reads were retrieved and assembled with Canu v2.2 [6]. Any prokaryotic sequences remaining at this stage were removed and the mitochondrial genome (48 kb) was recovered. The decontaminated draft genome was polished in three rounds using Illumina reads with Pilon v1.24 [9].
Complete (telomere-to-telomere) chromosomes (n = 133) were identified based on the presence of telomeric repeats at contig ends using the Telomere Identification Toolkit (tidk) [10], in combination with Geneious (https://www.geneious.com/). Genome completeness was estimated using BUSCO v5.5 using the conserved eukaryotic ortholog set (eukaryota_odb10, n=255) (Supplementary Table S12) [11].

Gene prediction, annotation and repeat analyses.
Eukaryotic genes in the CRY1a genome were predicted using BRAKER3 v3.0.8 [12], with transcriptomic data serving as evidence. Filtered paired-end Illumina RNA-seq reads were mapped to the genome using HISAT2 [13] in spliced alignment mode (--sensitive --no-mixed --no-discordant). The resulting BAM files (sorted and indexed) were used as hints to improve gene prediction accuracy. Genome completeness was assessed with BUSCO v5.5.0 [11] (Supplementary Table S12). Predicted protein-coding genes were annotated using KEGG KOALA (KEGG Orthology And Links Annotation) [14]. Metabolic pathways identified as complete by KOALA were subsequently validated through manual curation (provided in Supplementary Table S8). Transfer RNA (tRNA) genes in the nuclear genome were identified using ARAGORN (settings:-t -gcstd -w -i) [15] and tRNAscan-SE v2.0.2 with default settings [16].

Repetitive regions, including simple repeats and mobile genetic elements, were identified using a two-step approach combining *ab initio* prediction and homology-based searches. *Ab initio* detection was performed with RepeatModeler v2.0.3 [17], which incorporates RECON v1.08 [18], RepeatScout v1.0.6 [19], rmblast v2.11.0+, and TRF v4.09 [20]. Long terminal repeats (LTRs) were identified using RepeatModeler with the -LTRStruct option, invoking LtrHarvest [21] via GenomeTools v1.6.2 [22], and LTR_retriever v2.9.0 [23], along with its dependencies MAFFT v7.487 [24], CD-HIT v4.8.1 [25], and Ninja v0.95 [26]. The resulting

custom repeat library was combined with the Dfam v3.5 database (2021-10-08; https://dfam.org) for homology-based masking using RepeatMasker v4.1.2 (https://www.repeatmasker.org/).

Transcriptome sequencing and assembly.

Illumina paired-end RNA sequencing (2 × 150 bp, 350 bp insert size) was performed following poly(A) enrichment, with a target yield of 10 Gbp. Library preparation and sequencing were conducted at Novogene (Hong Kong, China). Raw RNA-seq reads were processed using the same quality control pipeline applied to the Illumina whole-genome shotgun (WGS) data. Curated paired-end reads were assembled into transcripts using MEGAHIT v1.2.9 [27] with the following settings: --min-count 2 --k-list 29,39,49,...149 (step size of 10). Protein-coding genes were predicted in assembled transcripts using Prodigal v2.6.3 [7].

Phylogenomic tree of cryptophytes.

A phylogenomic dataset comprising representative cryptophytes (n=16) and the outgroup lineages Glaucophyta (n=2) and Chloroplastida (n=4) was assembled using the standard PhyloFisher [28] workflow. In addition to the nine *Cryptista* taxa already included in PhyloFisherDatabase v1.0, we predicted and appended proteomes derived from the transcriptomes of *Tyrannomonas regina* RIE21 and *Limnogoniomonas* H4 (both sequenced in this study), as well as publicly available RNA-seq datasets from *Microheliella maris* (DRR333359), *Hemiarma marina* (DRR333360), and three katablepharid isolates (38W - SRX6831925; T4 - SRX6831936; T2 - SRX6831935). In all cases, raw paired-end RNA-seq reads were adapter- and quality-trimmed using bbduk.sh from the BBMap suite [29], assembled with Trinity [30], and translated into predicted proteins using TD2 [31]. All resulting proteomes were incorporated into the Phylofisher database, and homologs were identified via HMMER searches. Single-gene phylogenetic trees were generated following the Phylofisher pipeline: homologous sequences were aligned with MAFFT, filtered with PREQUAL and DIVVIER, trimmed using BMGE and trimAl, and phylogenetic trees were inferred with RAxML. Gene trees were manually curated using ParaSorter to remove paralogs, followed by re-alignment, trimming, and concatenation of the remaining orthologs (n = 224).

The final concatenated supermatrix assembled by Phylofisher [28] comprised 79,888 aligned amino acid positions with 64,781 distinct patterns, 45,631 parsimony-informative sites, 13,444 singletons, and 20,813 constant sites. A maximum likelihood tree was constructed using IQ-TREE2 v2.3.4 [32] with the substitution model Q.yeast+F+I+G4, selected by ModelFinder [33] according to the highest Bayesian Information Criterion (BIC) score. Branch support values

were generated with 1,000 ultrafast bootstrap support iterations and 1,000 SH-aLRT test replicates.

Recovery of giant virus genomes.

Initial discovery: During long-read sequencing of CRY1a (see Methods), a giant virus (667 kb) belonging to the phylum *Nucleocytoviricota* was detected in the same culture using geNomad v1.7.6 [34]. However, despite a median coverage of ~54×, the genome could not be confidently resolved due to the presence of exceptionally long terminal inverted repeats (TIRs) exceeding 100 kbp. To ensure that these repeats were genuine and not assembly artifacts, additional sequencing was performed on the viral fraction alone.

Targeted recovery: To recover complete viral genomes, a CRY1a culture (~200 mL) exhibiting signs of virus-induced collapse was subjected to serial filtration through 0.8 µm, 0.45 µm, and 0.1 µm filters (Minisart® Syringe Filter, PES). All filters were preserved in DNA/RNA Shield (Zymo, R1200) and stored at –80 °C. DNA was extracted from biomass on the 0.1 µm filter using the Quick-DNA™ HMW MagBead Kit (Zymo, D6060) following the manufacturer's protocol. Long-read sequencing was performed locally on a MinION™ Mk1B sequencing device (ONT) using the Rapid Sequencing Kit with barcoding (SQK-RBK114.24). The sample was run in duplicate (2x40 ng of input DNA) under two distinct barcodes on a FLO-MIN114 (R10.4.1) flow cell for 72 hours. Raw reads were basecalled using Guppy (v6.5.7) with the dna_r10.4.1_e8.2_400bps_sup.cfg model. Sequencing yielded 613,779 reads, totalling 2.52 Gbp with an N50 of 7.63 kb (Q ≥ 10).

Giant virus genome assembly: Basecalled long reads in FASTQ format were adapter- and barcode-trimmed using Porechop v0.2.4 [4]. Trimmed reads were assembled with Canu v2.2 [6] using default parameters (genomeSize=1m). The resulting assembly was filtered to retain contigs ≥10 kb (27.8 Mb, 107 contigs). Viral contigs were identified using geNomad v1.9.0 [34]. Terminal inverted repeats (TIRs) were fully resolved using Elloreas, a long-read-based extension tool that iteratively maps overhanging reads to contig ends, builds a consensus, and extends the sequence [35]. Elloreas was run in both orientations with a minimum of 20 supporting reads required for each extension iteration.

Coverage estimates: The trimmed long-read viral fraction dataset was mapped to the final giant virus genomes (excluding terminal inverted repeats) using minimap2 [8] with stringent parameters to account for the high sequence similarity between the two recovered viruses: -ax asm5 --eqx --secondary=no --score-N 0 --max-chain-skip 25 --max-chain-iter 400 --end-bonus 5 --min-occ-floor 100. SAMtools v1.9 [36] was used to sort, index and calculate per

base coverage. The median per-base coverage across the entire core genome (i.e., without TIRs) was calculated using an AWK script.

Expression analysis: Curated RNA-seq paired-end reads were mapped to the giant virus genomes assembled from the culture using HISAT2 [13], with the following custom parameters: --sensitive --no-mixed --no-discordant --no-spliced-alignment. The resulting BAM files were sorted and indexed using SAMtools v1.9 [36]. Read counts over predicted viral genes were obtained using BEDTools coverage [37] with the corresponding BED files. Although strictly localized regions of low-to-medium coverage were observed, they overlapped only a small portion of the predicted gene lengths, indicating an absence of viral gene expression, consistent with the lack of active infection at the time of RNA collection.

Phylogenomic tree of giant viruses.

Open reading frames (ORFs) for the two reconstructed giant viruses, GV1 and GV2, were predicted using Prodigal v2.6.3 in metagenomic mode [7]. The predicted proteomes, together with a curated reference set of 694 published viral genomes - comprising both well-characterised isolates and metagenome-assembled viruses - were scanned for seven conserved proteins (A32, SFII, RNAPL, PolB, TFIIB, TopoII, and VLTF3) previously validated for phylogenetic analysis across *Nucleocytoviricota [38]*. Marker proteins were identified using hmmsearch v3.3 (e-value <1e-3) [39] against two sets of HMM profiles: 1) profiles from ViralRecall (https://github.com/faylward/viralrecall) and 2) profiles from the GVOG database (https://github.com/faylward/ncldv_markersearch/tree/master/hmm). Recovered protein sequences were individually aligned for each marker using MAFFT v7.450 [24] in E-INS-i mode. Alignments were then trimmed with BMGE v2.0 [40] using the settings -g 0.5 -b 3 -m BLOSUM30. Trimmed alignments were concatenated using the catfasta2phyml.pl script (https://github.com/nylander/catfasta2phyml) with options -c -s -v -f. A maximum likelihood phylogenomic tree was inferred from the concatenated alignment using IQ-TREE2 v2.3.4 [32] with settings -B 1000 --alrt 1000 -m TEST. The final alignment comprised 696 sequences, 3095 columns, 3092 distinct patterns, 3004 parsimony-informative sites, 47 singleton sites, and 44 constant sites. The best-fitting evolutionary model, LG+F+I+G4, was selected by ModelFinder [33] based on the Bayesian Information Criterion (BIC).

Endogenous viral elements (EVE) identification and analysis.

A combination of complementary indicators was used to identify putative endogenous Polinton-like viruses (PLVs) and virophages in the CRY1a genome, including the detection of hallmark major capsid proteins (MCPs), shifts in GC content, and the presence of

characteristic flanking repeats, either direct or inverted. Gene prediction was first carried out using Prodigal v2.6.3 [7]. Resulting protein sequences were screened with hmmsearch (e-value ≤ 1e-3) [39] against a curated set of 86 hallmark PLV and virophage MCP HMM profiles (available via Zenodo: 10.5281/zenodo.14540677). Only hits covering at least 50% of both the HMM model and the protein sequence were retained. Genomic coordinates of MCP genes were extracted from the filtered results and formatted as BED files. GC content profiles were generated across all chromosomes using a custom script that utilised the *makewindows* and *nuc* subprograms from BEDTools [37], with a defined window size of 200 bp. These, along with the MCP gene coordinates, were loaded into Integrative Genomics Viewer (IGV) [41] for visualisation. Regions containing putative PLVs were readily identified by their distinct GC content and co-localisation with MCP genes. Each region was manually selected along with approximately 5 kb of flanking sequence on either side. Coordinates were exported as BED files and used to extract the corresponding sequences from the genome. These candidate PLV loci were subsequently curated in Geneious v9.1.3 (https://www.geneious.com). DotPlots were generated to visually detect terminal repeats - either direct (DRs) or inverted (TIRs). The boundaries of each PLV were refined by aligning terminal regions and identifying highly similar sequences in either the forward or reverse-complement orientation. Additionally, flanking sequences up to 20 bp upstream and downstream of each element were manually inspected for the presence of target site duplications (TSDs), which may arise during integration within the host genome.

PLV gene annotation: Predicted protein sequences were first scanned against several functional annotation databases, including the Kyoto Encyclopedia of Genes and Genomes (KEGG), Clusters of Orthologous Groups (COG), TIGRFAMs (The Institute for Genomic Research Protein Families), and the Protein Families database (PFAM) [14, 42–45]. In parallel, annotation was performed using InterProScan [44]. Due to the high proportion of unannotated proteins, each sequence was individually annotated using the HHpred web server (https://toolkit.tuebingen.mpg.de/), with searches performed against the following databases: PDB_mmCIF70_30_Mar (Protein Data Bank structures clustered at 70% identity), Pfam-A v37, UniProt-SwissProt-viral70_3_Nov_2021 (Viral subset of SwissProt clustered at 70% identity), NCBI Conserved Domains (CD v3.19). For large protein clusters (>10 sequences) that remained unannotated, additional sensitive searches were conducted using HHblits (https://toolkit.tuebingen.mpg.de/tools/hhblits) against the UniRef30_2023_02 database, before forwarding aligned regions to HHpred. Minor capsid protein (mCP) detection in *T. regina* PLV proteomes was performed with HHsearch v.2.0.15 [46] using custom profile HMM models, based on sequences previously confirmed as mCPs in 20 PLVs from rhizarian *Paulinella micropora* (WGS assembly accessions: BJOX01, WBZZ01)

[47]. *T. regina* PLV proteomes were scanned with HHsearch v.2.0.15[46] against these mCP profile HMMs.

Additionally, predicted PLV protein sequences were clustered into orthologous groups using MMseqs2 (easy-cluster mode) with ≥30% sequence identity and ≥80% alignment coverage. This facilitated the annotation of divergent proteins and the assignment of consensus labels. All relevant statistics, including exact genomic coordinates and repeat features, are provided in Supplementary Table S2. PLV annotations and orthologous group (OG) assignments are available in Supplementary Table S3.

Phylogenetic tree of PLV major capsid proteins (MCPs): Reference major capsid protein (MCP) sequences were curated to represent all known Polinton-like virus (PLV) groups and close relatives. Sequences were collected from multiple sources, including: 1) metagenomic and endogenous PLVs [47, 48], 2) isolate Gezel-14T [49], 3) isolate TsV-N1 [50], 4) metagenomic PLVs[51], 5) MMETSP transcriptomes [52], and 6) animal Maverick/Polintons from RepBase (https://www.girinst.org/repbase/; 2018). Additionally, MCPs from cryptophyte and ochrophyte genomes sequenced and assembled, or reassembled from existing data in our previous study [53] (10.5281/zenodo.14540677) were dereplicated to collapse identical sequences using MMseqs2 and included in the phylogenetic analysis. Highly divergent MCPs (e.g., from virophages and Metamonada) were excluded. MCPs from 12,199 high-quality PLVs [47] were dereplicated by clustering with MMseqs2 (80% coverage, 90% identity). An additional set of 1,506 curated MCPs with PLV cluster assignments was added from the same study, as well as dereplicated MMETSP transcripts and MCPs from isolated PLVs. Following multiple rounds of dereplication and manual curation, a total of 793 reference sequences were retained. Aside from MCPs detected here in *T. regina* PLVs (n=21), other Cryptophyte MCP sequences were included from: *Storeatula* sp. K-1488 (n=79), *Rhinomonas nottbecki* K-1855 (n=47), *Rhodomonas baltica* CCAP 979/9 (n=4), *Rhodomonas lacustris* NIVA 8/82 (n=400), *Cryptomonas pyrenoidifera* NIVA 2/81 (n=17), *Cryptomonas borealis* NIES-276 (n=15), *Neptunogoniomonas* (n=5), and *Guillardia theta* (n=2). A total of n=1,376 MCPs were included for phylogenetic analysis. All sequences were aligned using MAFFT (E-INS-i mode). This alignment (3,896 columns, 3649 distinct patterns, 2236 parsimony-informative, 824 singleton sites, 836 constant sites) was used to infer a maximum-likelihood tree in IQ-TREE2 (settings: -m TEST --perturb 0.2 --nstop 500 -B 1000 --alrt 1000) using ModelFinder-selected model Q.pfam+F+G. Phylogenetic clusters were annotated according to pre-defined MCP references [47].

Recovery of Tyrannovirus-like contigs from assembled metagenomes

Several freshwater metagenomic assemblies were gathered from prior publications [54–59]. Contigs were filtered at ≥10 kb and mapped to the core giant viral genomes (i.e., excluding terminal inverted repeats) using Minimap2 v2.29 with the following parameters: a (SAM output), and -x asm20. Unmapped reads were filtered out with samtools view -F 4, and alignments were sorted using samtools sort. Raw per-base coverage was calculated from the sorted BAM file using pysam (https://github.com/pysam-developers/pysam), counting mapped reads at each genomic position. Coverage was not normalised and represents the unadjusted read depth across the reference genome.

Fragment recruitment analyses.

To quantify the RPKM (Reads Per Kilobase per Million mapped reads) of giant viruses in global and time-series metagenomic datasets, we used CoverM v0.6.1 [60]. RPKM values were calculated from the initial CoverM outputs (-m covered_fraction count) using a custom R script, based on the formula: RPKM = (numReads) / (geneLength / 1,000) × (total number of reads / 1,000,000). Viral genomes were considered present in a sample if they had a covered fraction greater than 0.5; otherwise, their RPKM was set to zero. Coverage results are summarised in Supplementary Table S4.

Phylogenetic analysis of small ribosomal rRNA sequences.

A total of nine 18S rRNA sequences (length > 1500bp) were identified in the *Tyrannomonas* genome (https://ftp.ebi.ac.uk/pub/databases/Rfam/tools/rfam_scan-1.0.pl). At 99% identity, these sequences were dereplicated to a single representative using CD-HIT [25]. All cryptophyte 18S rRNA sequences from SILVA 138.1 [61], PR2 [62], and the EUKARYOME database [63] were collected. Additionally, 18S rRNA sequences (length > 800bp) were gathered from previous freshwater metagenomic assemblies and publications [3, 54, 55, 64, 65] and compared locally using MMseqs2 [66] to a combined database of SILVA 138.1, PR2, and EUKARYOME. Sequences with >90% identity and e-value below 1e-3 to database cryptophyte sequences were retained. These were submitted to online SILVA classification to capture *bona fide* cryptophyte sequences. Sequences not identified as cryptophytes were removed and the remaining sequences were dereplicated at 98% nucleotide identity. Chlorophyte sequences (n=26) were used as an outgroup for the phylogenetic analysis. The final collection of 257 sequences represents the broad diversity of cryptophyte 18S rRNA sequences. Alignments were created using mafft-linsi [24] and maximum-likelihood phylogenetic trees were constructed using Iqtree2 v.2.3.6 with ultra-fast bootstraps, SH-aLRT tests and automatic model selection using ModelFinder according to the Bayesian

Information Criterion (-B 1000 --alrt 1000 -m MFP) [32, 33, 67]. All sequences, alignments, and phylogenetic trees are publicly available in Zenodo (10.5281/zenodo.15210452).

Phylogenetic tree of rhodopsins.

Protein sequences predicted with BRAKER [12, 68] were screened for candidate rhodopsins using hmmsearch (e-value > 1e-3) against HMM profiles for Type-1, Type-3, and heliorhodopsins as described previously [69]. All candidate sequences were compared to a curated reference database of rhodopsins using MMseqs2 [66] to identify homologs. Multiple sequence alignments for each candidate were generated with MAFFT [24] in L-INS-i mode and used as input for Polyphobius to predict transmembrane helices [70]. Only sequences with seven predicted transmembrane helices and a conserved lysine (K) residue in TM7 were retained.

A total of 201 confirmed rhodopsin sequences recovered in this study were dereplicated using MMseqs2 [66] (easy-cluster mode; 100% coverage, 100% identity) and combined with a previously published dataset of 2,199 rhodopsins [69] (Supplementary Table S13). The combined set was aligned using MAFFT (L-INS-i) [24]. A maximum-likelihood phylogenetic tree was constructed using IQ-TREE2 v2.3.4 [32] based on the resulting alignment. The best-fit substitution model, Q.pfam+G4, was selected using ModelFinder [33] based on the Bayesian Information Criterion (BIC). Branch support was assessed with 1,000 ultrafast bootstrap replicates, and SH-aLRT tests. The final alignment included 2,265 sequences and 4,390 columns, comprising 3,848 distinct patterns, 2,322 parsimony-informative sites, 881 singletons, and 1,187 constant sites.

Supplementary Results

Gene content and metabolic characteristics.

Based on gene annotations, *T. regina* has a streamlined yet functional metabolic and cellular profile, adapted to a lifestyle reliant on bacterial prey. Pathways involved in central carbohydrate metabolism are complete, including glycolysis, gluconeogenesis, the pentose phosphate pathway, and PRPP biosynthesis (Supplementary Table S8). The TCA cycle appears mostly complete in the nuclear genome, although some subunits of succinate dehydrogenase are encoded in the mitochondrial genome. Similarly, a typical eukaryotic-like electron transport chain is present and is segregated between the nuclear and the mitochondrial genome. In terms of lipid metabolism, the organism has a complete pathway for fatty acid elongation and nearly complete modules for ketone body biosynthesis and

triacylglycerol biosynthesis, both missing only the terminal enzyme acetoacetate decarboxylase. These capacities suggest the ability to elongate and store lipids, and to utilise intermediates such as acetoacetate and 3-hydroxybutyrate. Importantly, it also possesses the enzymatic machinery for β-oxidation, enabling the degradation of fatty acids for energy—likely derived from ingested bacterial lipids. No evidence of autotrophic carbon fixation was found, as the Calvin cycle is absent. Additionally, *T. regina* lacks pathways for *de novo* nucleotide biosynthesis and for the biosynthesis of most amino acids. This implies a reliance on environmental or prey-derived sources for these metabolites, consistent with a bacterivorous lifestyle that supplies amino acids, nucleotides, vitamins, and cofactors (Supplementary Table S8). Despite these metabolic reductions, *T. regina* retains the essential components of core cellular processes. It encodes a complete eukaryotic-type ribosome, RNA polymerase, and a full set of aminoacyl-tRNA synthetases, supporting transcription and translation. Its DNA replication machinery is largely intact, including a complete pre-replication complex and primase, although some DNA polymerase subunits are missing or unannotated. Moreover, it encodes a functional spliceosome, indicating the ability to process pre-mRNA via intron removal, and a proteasome (20S, 26S) for regulated protein turnover. The presence of actins, tubulins, and actin-related proteins suggests a structured cytoskeleton that supports cell shape and intracellular transport and several cathepsins (A, B, C, D, F, L, and X), many of which are implicated in the phagocytic process [71] (Supplementary Table S8). Moreover, we found at least eight rhodopsins (Type-1) in the genome, three of which appeared to be *bona fide* proton-pumps (Supplementary Table S13 and Supplementary Figure S11) that may be involved either in sensory or photoheterotrophic roles. Five type-1 rhodopsins did not possess a retinal-binding lysine so may have been exapted for other as yet unknown functions. It has been postulated that such rhodopsins may be involved in nitrogen metabolism [72]. No heliorhodopsins were found in the *Tyrannomonas* genome. See more details below on rhodopsins.

Cryptista rhodopsins.

Rhodopsins are membrane proteins with seven transmembrane helices encapsulating a retinal chromophore which makes them photoreceptive [73, 74]. Their ability to sense photons and alter protein conformations allows them to integrate themselves in distinct light-sensitive biological activities such as outward proton translocation to generate a proton gradient (photoheterotrophy), as channels for cations or anions, or even function as inward proton pumps whose functional implications still remain unclear. They may also serve sensory functions, interacting with downstream proteins and triggering a cascade. Another type of rhodopsins (heliorhodopsins) have been discovered that have a membrane orientation opposite to classical Type-1 rhodopsins [75]. While Type-1 rhodopsins (with

proton-pumping rhodopsins being the most common) are widely distributed across all domains of life, heliorhodopsins appear restricted exclusively to eukaryotes and monoderms [76, 77].

We detected eight rhodopsins in the *T. regina* genome. All these were Type-1 rhodopsins and no heliorhodopsins were found in this genome (Supplementary Table S13). Five of these rhodopsins did not encode the classical retinal binding lysine in the seventh transmembrane helix and had arginine instead. Such rhodopsins have been described in cryptophytes before(e.g., in *Guillardea theta*) and are referred to as Rh-noKs [72]. They do not appear to be able to bind the retinal chromophore [78]. Similar rhodopsins (e.g., some FArhodopsins (flotillin-associated rhodopsins)) have also been reported before from freshwater and marine bacteria [79] and retinal-binding can be restored by reengineering a lysine residue at the same location [78]. We also examined rhodopsins in all other available cryptophyte genomes and three additional katablepharid transcriptomes. In total (including *T. regina*), these genomes and transcriptomes yielded 201 *bona fide* rhodopsin sequences. Of these, 54 were Alt-Rhodopsins [69], lacking the conserved arginine-82 residue in the third transmembrane helix and use another amino acid instead (27 K-type using lysine, 17 H-type using histidine, and a small number of others, Supplementary Table S13). The canonical Type-1 rhodopsins in particular appear greatly expanded in cryptophytes (e.g., >50 rhodopsins were found in *Storeatula* and *Rhinomonas* genomes). The heterotrophic *Neptunogoniomonas avonlea* genome contained only three rhodopsins (all Type-1). In comparison, very few heliorhodopsins were found, the maximum being seven in *Storeatula*. Moreover, in *Storeatula* at least seven rhodopsin sequences were completely identical, indicating a recent gene duplication event. A phylogenetic analysis of all rhodopsins retrieved revealed multiple distinct branches of cryptista rhodopsins (Supplementary Figure S11). These appear to be of different types 1) those shared by both goniomonads and cryptophytes and 2) those comprising solely cryptophytes. Both have been observed in a prior study [69]. However, a third strongly supported branch comprising only three H-type rhodopsins from *T. regina* was also recovered (Supplementary Figure S11). In a recent study, such lysine-less rhodopsins from *Guillardea theta* (Rh-noKs), showed highly reduced expression in N-depletion conditions, and it was postulated that they have a role in nitrogen homeostasis but the mechanisms remain unknown [72]. A similar protein in yeast (Hsp30) has been implicated in the regulation of the plasma membrane H+ -ATPase [80]. These divergent branches of rhodopsins found in cryptophyte genomes suggest a complex evolutionary history of multiple independent acquisitions, gene duplications, and lack of retinal-binding ability in some lineages, likely leading to other biological roles.

Supplementary References

1. Grujcic V, Nuy JK, Salcher MM, et al. Cryptophyta as major bacterivores in freshwater summer plankton. *ISME J* 2018;**12**:1668–81. https://doi.org/10.1038/s41396-018-0057-5
2. Piwosz K, Mukherjee I, Salcher MM, et al. CARD-FISH in the sequencing era: Opening a new universe of protistan ecology. *Front Microbiol* 2021;**12**:640066. https://doi.org/10.3389/fmicb.2021.640066
3. Šimek K, Mukherjee I, Szöke-Nagy T, et al. Cryptic and ubiquitous aplastidic cryptophytes are key freshwater flagellated bacterivores. *ISME J* 2023;**17**:84–94. https://doi.org/10.1038/s41396-022-01326-4
4. Wick RR, Judd LM, Gorrie CL, et al. Completing bacterial genome assemblies with multiplex MinION sequencing. *Microb Genom* 2017;**3**:e000132. https://doi.org/10.1099/mgen.0.000132
5. Kolmogorov M, Yuan J, Lin Y, et al. Assembly of long, error-prone reads using repeat graphs. *Nat Biotechnol* 2019;**37**:540–6. https://doi.org/10.1038/s41587-019-0072-8
6. Koren S, Walenz BP, Berlin K, et al. Canu: scalable and accurate long-read assembly via adaptive k-mer weighting and repeat separation. *Genome Res* 2017;**27**:722–36. https://doi.org/10.1101/gr.215087.116
7. Hyatt D, Chen G-L, Locascio PF, et al. Prodigal: prokaryotic gene recognition and translation initiation site identification. *BMC Bioinformatics* 2010;**11**:119. https://doi.org/10.1186/1471-2105-11-119
8. Li H. Minimap2: pairwise alignment for nucleotide sequences. *Bioinformatics* 2018;**34**:3094–100. https://doi.org/10.1093/bioinformatics/bty191
9. Walker BJ, Abeel T, Shea T, et al. Pilon: an integrated tool for comprehensive microbial variant detection and genome assembly improvement. *PLoS One* 2014;**9**:e112963. https://doi.org/10.1371/journal.pone.0112963

10. Brown MR, Manuel Gonzalez de La Rosa P, Blaxter M. Tidk: A toolkit to rapidly identify telomeric repeats from genomic datasets. *Bioinformatics* 2025;**41**:btaf049. https://doi.org/10.1093/bioinformatics/btaf049
11. Manni M, Berkeley MR, Seppey M, et al. BUSCO: Assessing Genomic Data Quality and Beyond. *Curr Protoc* 2021;**1**:e323. https://doi.org/10.1002/cpz1.323
12. Gabriel L, Brůna T, Hoff KJ, et al. BRAKER3: Fully automated genome annotation using RNA-seq and protein evidence with GeneMark-ETP, AUGUSTUS, and TSEBRA. *Genome Res* 2024;**34**:769–77. https://doi.org/10.1101/gr.278090.123
13. Kim D, Paggi JM, Park C, et al. Graph-based genome alignment and genotyping with HISAT2 and HISAT-genotype. *Nat Biotechnol* 2019;**37**:907–15. https://doi.org/10.1038/s41587-019-0201-4
14. Kanehisa M, Sato Y, Morishima K. BlastKOALA and GhostKOALA: KEGG Tools for Functional Characterization of Genome and Metagenome Sequences. *J Mol Biol* 2016. https://doi.org/10.1016/j.jmb.2015.11.006
15. Laslett D, Canback B. ARAGORN, a program to detect tRNA genes and tmRNA genes in nucleotide sequences. *Nucleic Acids Res* 2004;**32**:11–6. https://doi.org/10.1093/nar/gkh152
16. Chan PP, Lowe TM. tRNAscan-SE: Searching for tRNA Genes in Genomic Sequences. *Methods Mol Biol* 2019;**1962**:1–14. https://doi.org/10.1007/978-1-4939-9173-0_1
17. Flynn JM, Hubley R, Goubert C, et al. RepeatModeler2 for automated genomic discovery of transposable element families. *Proc Natl Acad Sci U S A* 2020;**117**:9451–7. https://doi.org/10.1073/pnas.1921046117
18. Bao Z, Eddy SR. Automated de novo identification of repeat sequence families in sequenced genomes. *Genome Res* 2002;**12**:1269–76. https://doi.org/10.1101/gr.88502
19. Price AL, Jones NC, Pevzner PA. De novo identification of repeat families in large genomes. *Bioinformatics* 2005;**21 Suppl 1**:i351–8. https://doi.org/10.1093/bioinformatics/bti1018
20. Benson G. Tandem repeats finder: a program to analyze DNA sequences. *Nucleic*

Acids Res 1999;**27**:573–80. https://doi.org/10.1093/nar/27.2.573

21. Ellinghaus D, Kurtz S, Willhoeft U. LTRharvest, an efficient and flexible software for de novo detection of LTR retrotransposons. *BMC Bioinformatics* 2008;**9**:18. https://doi.org/10.1186/1471-2105-9-18
22. Gremme G, Steinbiss S, Kurtz S. GenomeTools: a comprehensive software library for efficient processing of structured genome annotations. *IEEE/ACM Trans Comput Biol Bioinform* 2013;**10**:645–56. https://doi.org/10.1109/TCBB.2013.68
23. Ou S, Jiang N. LTR_retriever: A Highly Accurate and Sensitive Program for Identification of Long Terminal Repeat Retrotransposons. *Plant Physiol* 2018;**176**:1410–22. https://doi.org/10.1104/pp.17.01310
24. Katoh K, Standley DM. MAFFT multiple sequence alignment software version 7: improvements in performance and usability. *Mol Biol Evol* 2013;**30**:772–80. https://doi.org/10.1093/molbev/mst010
25. Li W, Godzik A. Cd-hit: a fast program for clustering and comparing large sets of protein or nucleotide sequences. *Bioinformatics* 2006;**22**:1658–9. https://doi.org/10.1093/bioinformatics/btl158
26. Wheeler TJ. Large-Scale Neighbor-Joining with NINJA. *Algorithms in Bioinformatics* 2009 Springer Berlin Heidelberg, 2009. 375–89
27. Li D, Luo R, Liu C-M, et al. MEGAHIT v1.0: A fast and scalable metagenome assembler driven by advanced methodologies and community practices. *Methods* 2016;**102**:3–11. https://doi.org/10.1016/j.ymeth.2016.02.020
28. Jones RE, Tice AK, Eliáš M, et al. Create, analyze, and visualize phylogenomic datasets using PhyloFisher. *Curr Protoc* 2024;**4**:e969. https://doi.org/10.1002/cpz1.969
29. Bushnell B. BBMap: A fast, accurate, splice-aware aligner. Lawrence Berkeley National Lab. (LBNL), Berkeley, CA (United States), 2014.
30. Grabherr MG, Haas BJ, Yassour M, et al. Full-length transcriptome assembly from RNA-Seq data without a reference genome. *Nat Biotechnol* 2011;**29**:644–52. https://doi.org/10.1038/nbt.1883

31. Mao A, Ji HJ, Haas B, et al. TD2: finding protein coding regions in transcripts. *bioRxiv* 2025 2025.
32. Minh BQ, Schmidt HA, Chernomor O, et al. IQ-TREE 2: New Models and Efficient Methods for Phylogenetic Inference in the Genomic Era. *Mol Biol Evol* 2020;**37**:1530–4. https://doi.org/10.1093/molbev/msaa015
33. Kalyaanamoorthy S, Minh BQ, Wong TKF, et al. ModelFinder: fast model selection for accurate phylogenetic estimates. *Nat Methods* 2017;**14**:587–9. https://doi.org/10.1038/nmeth.4285
34. Camargo AP, Roux S, Schulz F, et al. Identification of mobile genetic elements with geNomad. *Nat Biotechnol* 2024;**42**:1303–12. https://doi.org/10.1038/s41587-023-01953-y
35. Logacheva MD, Schelkunov MI, Fesenko AN, et al. Mitochondrial genome of *Fagopyrum esculentum* and the genetic diversity of extranuclear genomes in buckwheat. *Plants* 2020;**9**:618. https://doi.org/10.3390/plants9050618
36. Li H, Handsaker B, Wysoker A, et al. The Sequence Alignment/Map format and SAMtools. *Bioinformatics* 2009;**25**:2078–9. https://doi.org/10.1093/bioinformatics/btp352
37. Quinlan AR. BEDTools: The Swiss-Army Tool for Genome Feature Analysis. *Curr Protoc Bioinformatics* 2014;**47**:11.12.1–34. https://doi.org/10.1002/0471250953.bi1112s47
38. Aylward FO, Moniruzzaman M, Ha AD, et al. A phylogenomic framework for charting the diversity and evolution of giant viruses. *PLoS Biol* 2021;**19**:e3001430. https://doi.org/10.1371/journal.pbio.3001430
39. Potter SC, Luciani A, Eddy SR, et al. HMMER web server: 2018 update. *Nucleic Acids Res* 2018;**46**:W200–4. https://doi.org/10.1093/nar/gky448
40. Criscuolo A, Gribaldo S. BMGE (Block Mapping and Gathering with Entropy): a new software for selection of phylogenetic informative regions from multiple sequence alignments. *BMC Evol Biol* 2010;**10**:210. https://doi.org/10.1186/1471-2148-10-210
41. Robinson JT, Thorvaldsdóttir H, Winckler W, et al. Integrative genomics viewer. *Nat*

Biotechnol 2011;**29**:24–6. https://doi.org/10.1038/nbt.1754

42. Galperin MY, Makarova KS, Wolf YI, et al. Expanded microbial genome coverage and improved protein family annotation in the COG database. *Nucleic Acids Res* 2015;**43**:D261–9. https://doi.org/10.1093/nar/gku1223
43. Haft DH, Selengut JD, White O. The TIGRFAMs database of protein families. *Nucleic Acids Res* 2003;**31**:371–3. https://doi.org/10.1093/nar/gkg128
44. Jones P, Binns D, Chang H-Y, et al. InterProScan 5: genome-scale protein function classification. *Bioinformatics* 2014;**30**:1236–40. https://doi.org/10.1093/bioinformatics/btu031
45. El-Gebali S, Mistry J, Bateman A, et al. The Pfam protein families database in 2019. *Nucleic Acids Res* 2019;**47**:D427–32. https://doi.org/10.1093/nar/gky995
46. Söding J. Protein homology detection by HMM–HMM comparison. *Bioinformatics* 2005;**21**:951–60. https://doi.org/10.1093/bioinformatics/bti125
47. Bellas C, Hackl T, Plakolb M-S, et al. Large-scale invasion of unicellular eukaryotic genomes by integrating DNA viruses. *Proc Natl Acad Sci U S A* 2023;**120**:e2300465120. https://doi.org/10.1073/pnas.2300465120
48. Bellas CM, Sommaruga R. Polinton-like viruses are abundant in aquatic ecosystems. *Microbiome* 2021;**9**:13. https://doi.org/10.1186/s40168-020-00956-0
49. Roitman S, Rozenberg A, Lavy T, et al. Isolation and infection cycle of a polinton-like virus virophage in an abundant marine alga. *Nat Microbiol* 2023;**8**:332–46. https://doi.org/10.1038/s41564-022-01305-7
50. Pagarete A, Grébert, Théophile, Stepanova O, et al. Tsv-N1: A Novel DNA Algal Virus that Infects *Tetraselmis striata*. *Viruses* 2015;**7**:3937–53. https://doi.org/10.3390/v7072806
51. Yutin N, Shevchenko S, Kapitonov V, et al. A novel group of diverse Polinton-like viruses discovered by metagenome analysis. *BMC Biol* 2015;**13**:95. https://doi.org/10.1186/s12915-015-0207-4
52. Keeling PJ, Burki F, Wilcox HM, et al. The Marine Microbial Eukaryote Transcriptome

Sequencing Project (MMETSP): illuminating the functional diversity of eukaryotic life in the oceans through transcriptome sequencing. *PLoS Biol* 2014;**12**:e1001889. https://doi.org/10.1371/journal.pbio.1001889

53. Bulzu P-A, Henriques Vieira H, Ghai R. Lineage-specific expansions of polinton-like viruses in photosynthetic cryptophytes. *Microbiome* 2025;**13**:154. https://doi.org/10.1186/s40168-025-02148-0
54. Kavagutti VS, Bulzu P-A, Chiriac CM, et al. High-resolution metagenomic reconstruction of the freshwater spring bloom. *Microbiome* 2023;**11**:15. https://doi.org/10.1186/s40168-022-01451-4
55. Kavagutti VS, Andrei A-Ş, Mehrshad M, et al. Phage-centric ecological interactions in aquatic ecosystems revealed through ultra-deep metagenomics. *Microbiome* 2019;**7**:135. https://doi.org/10.1186/s40168-019-0752-0
56. Chiriac M-C, Bulzu P-A, Andrei A-S, et al. Ecogenomics sheds light on diverse lifestyle strategies in freshwater CPR. *Microbiome* 2022;**10**:84. https://doi.org/10.1186/s40168-022-01274-3
57. Garner RE, Kraemer SA, Onana VE, et al. A genome catalogue of lake bacterial diversity and its drivers at continental scale. *Nat Microbiol* 2023;**8**:1920–34. https://doi.org/10.1038/s41564-023-01435-6
58. Mehrshad M, Salcher MM, Okazaki Y, et al. Hidden in plain sight-highly abundant and diverse planktonic freshwater Chloroflexi. *Microbiome* 2018;**6**:176. https://doi.org/10.1186/s40168-018-0563-8
59. Andrei A-Ş, Salcher MM, Mehrshad M, et al. Niche-directed evolution modulates genome architecture in freshwater Planctomycetes. *ISME J* 2019;**13**:1056–71. https://doi.org/10.1038/s41396-018-0332-5
60. Aroney STN, Newell RJP, Nissen JN, et al. CoverM: read alignment statistics for metagenomics. *Bioinformatics* 2025;**41**:btaf147. https://dx.doi.org/10.1093/bioinformatics/btaf147
61. Quast C, Pruesse E, Yilmaz P, et al. The SILVA ribosomal RNA gene database project:

improved data processing and web-based tools. *Nucleic Acids Res* 2013;**41**:D590–6. https://doi.org/10.1093/nar/gks1219

62. Guillou L, Bachar D, Audic S, et al. The Protist Ribosomal Reference database (PR2): a catalog of unicellular eukaryote small sub-unit rRNA sequences with curated taxonomy. *Nucleic Acids Res* 2013;**41**:D597–604. https://doi.org/10.1093/nar/gks1160
63. Tedersoo L, Hosseyni Moghaddam MS, Mikryukov V, et al. EUKARYOME: the rRNA gene reference database for identification of all eukaryotes. *Database (Oxford)* 2024;**2024**. https://doi.org/10.1093/database/baae043
64. Boukheloua R, Mukherjee I, Park H, et al. Global freshwater distribution of Telonemia protists. *ISME J* 2024;**18**:wrae177. https://doi.org/10.1093/ismejo/wrae177
65. Shalchian-Tabrizi K, Bråte J, Logares R, et al. Diversification of unicellular eukaryotes: cryptomonad colonizations of marine and fresh waters inferred from revised 18S rRNA phylogeny. *Environ Microbiol* 2008;**10**:2635–44. https://doi.org/10.1111/j.1462-2920.2008.01685.x
66. Steinegger M, Söding J. MMseqs2 enables sensitive protein sequence searching for the analysis of massive data sets. *Nat Biotechnol* 2017;**35**:1026–8. https://doi.org/10.1038/nbt.3988
67. Hoang DT, Chernomor O, von Haeseler A, et al. UFBoot2: Improving the Ultrafast Bootstrap Approximation. *Mol Biol Evol* 2018;**35**:518–22. https://doi.org/10.1093/molbev/msx281
68. Brůna T, Hoff KJ, Lomsadze A, et al. BRAKER2: automatic eukaryotic genome annotation with GeneMark-EP+ and AUGUSTUS supported by a protein database. *NAR Genom Bioinform* 2021;**3**:lqaa108. https://doi.org/10.1093/nargab/lqaa108
69. Bulzu P-A, Kavagutti VS, Andrei A-S, et al. The Evolutionary Kaleidoscope of Rhodopsins. *mSystems* 2022;**7**:e0040522. https://doi.org/10.1128/msystems.00405-22
70. Käll L, Krogh A, Sonnhammer ELL. An HMM posterior decoder for sequence feature prediction that includes homology information. *Bioinformatics* 2005;**21 Suppl 1**:i251–7. https://doi.org/10.1093/bioinformatics/bti1014

71. Flannagan RS, Jaumouillé V, Grinstein S. The cell biology of phagocytosis. *Annu Rev Pathol* 2012;**7**:61–98. https://doi.org/10.1146/annurev-pathol-011811-132445

72. Konno M, Yamauchi Y, Inoue K, et al. Expression analysis of microbial rhodopsin-like genes in *Guillardia theta*. *PLoS One* 2020;**15**:e0243387. https://doi.org/10.1371/journal.pone.0243387

73. Rozenberg A, Inoue K, Kandori H, et al. Microbial Rhodopsins: The Last Two Decades. *Annu Rev Microbiol* 2021;**75**:427–47. https://doi.org/10.1146/annurev-micro-031721-020452

74. Nagata T, Inoue K. Rhodopsins at a glance. *J Cell Sci* 2021;**134**. https://doi.org/10.1242/jcs.258989

75. Pushkarev A, Inoue K, Larom S, et al. A distinct abundant group of microbial rhodopsins discovered using functional metagenomics. *Nature* 2018;**558**:595–9. https://doi.org/10.1038/s41586-018-0225-9

76. Bulzu P-A, Kavagutti VS, Chiriac M-C, et al. Heliorhodopsin Evolution Is Driven by Photosensory Promiscuity in Monoderms. *mSphere* 2021;**6**:e0066121. https://doi.org/10.1128/mSphere.00661-21

77. Flores-Uribe J, Hevroni G, Ghai R, et al. Heliorhodopsins are absent in diderm (Gram-negative) bacteria: Some thoughts and possible implications for activity: Heliorhodopsins are absent in diderms. *Environ Microbiol Rep* 2019;**11**:419–24. https://doi.org/10.1111/1758-2229.12730

78. Yamauchi Y, Konno M, Yamada D, et al. Engineered functional recovery of microbial rhodopsin without retinal-binding lysine. *Photochem Photobiol* 2019;**95**:1116–21. https://doi.org/10.1111/php.13114

79. Haro-Moreno JM, López-Pérez M, Alekseev A, et al. Flotillin-associated rhodopsin (FArhodopsin), a widespread paralog of proteorhodopsin in aquatic bacteria with streamlined genomes. *mSystems* 2023;**8**:e0000823. https://doi.org/10.1128/msystems.00008-23

80. Piper PW, Ortiz-Calderon C, Holyoak C, et al. Hsp30, the integral plasma membrane

heat shock protein of *Saccharomyces cerevisiae*, is a stress-inducible regulator of plasma membrane H(+)-ATPase. *Cell Stress Chaperones* 1997;**2**:12–24. https://doi.org/10.1379/1466-1268(1997)002<0012:htipmh>2.3.co;2
